# Understanding the Potential *In Vitro* Modes of Action of Bis(β‐diketonato) Oxovanadium(IV) Complexes

**DOI:** 10.1002/cmdc.202100152

**Published:** 2021-05-26

**Authors:** Baris Sergi, Ipek Bulut, Ying Xia, Zoë A. E. Waller, Yasemin Yildizhan, Ceyda Acilan, Rianne M. Lord

**Affiliations:** ^1^ School of Chemistry University of East Anglia Norwich Research Park NR4 7TJ Norwich UK; ^2^ Gradute School of Health Sciences Koç University 34450 Sariyer Istanbul Turkey; ^3^ School of Pharmacy University of East Anglia Norwich Research Park NR4 7TJ Norwich UK; ^4^ School of Pharmacy UCL 29–39 Brunswick Square WC1E 6BT London UK; ^5^ Genetic Engineering and Biotechnology Institute TUBITAK 41470 Kocaeli Turkey; ^6^ School of Medicine Koç University 34450 Sariyer Istanbul Turkey; ^7^ Research Center for Translational Medicine Koç University 34450 Sariyer Istanbul Turkey

**Keywords:** Apoptosis, β-diketonate complexes, Bioinorganic Chemistry, DNA interactions, Vanadium complexes

## Abstract

To understand the potential *in vitro* modes of action of bis(β‐diketonato) oxovanadium(IV) complexes, nine compounds of varying functionality have been screened using a range of biological techniques. The antiproliferative activity against a range of cancerous and normal cell lines has been determined, and show these complexes are particularly sensitive against the lung carcinoma cell line, A549. Annexin V (apoptosis) and Caspase‐3/7 assays were studied to confirm these complexes induce programmed cell death. While gel electrophoresis was used to determine DNA cleavage activity and production of reactive oxygen species (ROS), the Comet assay was used to determine induced genomic DNA damage. Additionally, Förster resonance energy transfer (FRET)‐based DNA melting and fluorescent intercalation displacement assays have been used to determine the interaction of the complexes with double strand (DS) DNA and to establish preferential DNA base‐pair binding (AT versus GC).

## Graphical abstract

**Outstanding oxovanadium**! Functionalized bis(β‐diketonato) oxovanadium(IV) complexes exhibiting increased cytotoxicity towards lung cancer cells are reported. The compounds show interactions with genomic DNA, a preference for A‐T binding and high levels of DNA fragmentation. Importantly, they generate high levels of reactive oxygen species and undergo apoptosis, which is predicted to be the main mode of cell death.

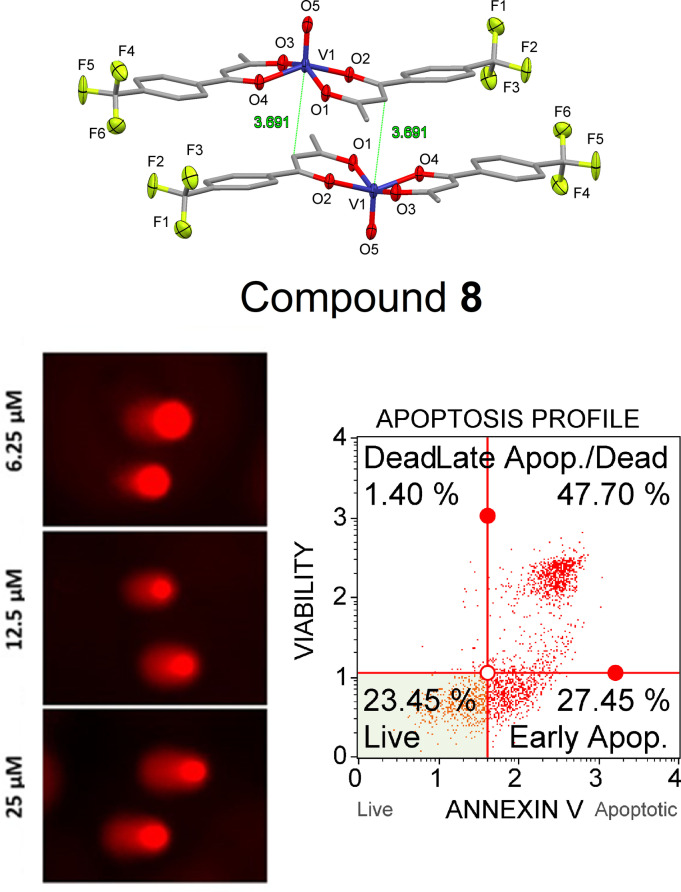

## Introduction

Although the anticancer properties of inorganic compounds appeared in 1930,^[1]^ the first indications for the antineoplastic effects of vanadium salts were not reported until 1965,^[2]^ and more in depth studies were not conducted until 1980–1990s. Sabbioni et al. have since reported on the vanadium metabolism and the cytotoxicity of vanadium compounds.[^3^, ^4^] In particular, the morphology transformations in mouse embryo after treatment with ammonium vanadate (V^V^) or vanadyl sulfate (V^IV^) salts has been studied, and it was noted that treatment with V^V^ alone, or in combination with diethylmaleate (DEM; a cellular glutathione (GSH)‐depleting agent), reduces the complex from V^V^ to V^IV^ (measured by EPR spectroscopy). Although V^IV^‐compounds show lower toxicity in the same cell line,[^4^, ^5^] it was later reported that vanadyl show no cytotoxic effects, whilst vanadate and pervanadate strongly inhibited cell development.^[6]^

Since the discovery of vanadium bromoperoxidase, a vanadium(V)‐containing enzyme in marine algae,[^7^, ^8^] the importance of vanadium in humans and diet has been an important research topic.^[9]^ In the early 1980s, vanadium was reported to act as an insulin mimic and was able to normalize diabetic rats.[^10^, ^11^] Since these reports, there has been a plethora of research on V‐compounds for their insulin‐like effects,[^12^, ^13^, ^14^, ^15^, ^16^, ^17^, ^18^, ^19^, ^20^] with groups studying their importance in different cell culture types.[^21^, ^22^] Vanadium(V) was shown to stimulate glucose uptake and glucose oxidation,[^23^, ^24^] and it was also postulated to inhibit tyrosine phosphate and bind to growth factors relating to cell proliferation.^[25]^

Over a decade later, bis(4,7‐dimethyl‐1,10‐phenanthroline) sulfato oxovanadium(IV) (Metvan) was discovered, and to the best of our knowledge, it remains the most promising V^IV^ anticancer compound to be reported.^[26]^ It exhibits nanomolar potency, and induces apoptosis in human leukemia cells, multiple myeloma cells and solid tumour cells (breast, glioblastoma, ovarian, prostate and testicular). It has been noted that the apoptosis is also associated with a loss of mitochondrial transmembrane and a depletion of GSH. Not only does this compound show high *in vitro* cytotoxicity, *in vivo* studies show no acute or subacute toxicity at 12.5–50 mg/kg.^[26]^

Since the discovery of Metvan, several research groups have studied V^IV^ complexes, showing they are better tolerated, more potent and have better cancer selectivity then clinical Pt(II) drugs.[^27^, ^28^] In 2018, Ni et al. produced a small library of mixed ligand oxidovanadium(IV) complexes with polycarboxylates and *N*‐heterocyclic ligands, with one compound showing high cytotoxicity, cell cycle S‐, or G_2_/M‐phase arrest and cell death by apoptosis.^[29]^ More recently, in 2020, Ribeiro et al. highlighted a range of VO(L) complexes (L=tridentate amino acid–pyridyl–phenol ligand), however, the complexes only exhibit moderate cytotoxicity values.^[30]^ One V^IV^‐compound exhibited increased late apoptosis when compared to the analogous Cu^II^ complex, and it was able to nick and cleave plasmid DNA. Reports on non‐oxidovanadium(IV) complexes have also emerged, with compounds exhibiting increased cytotoxicity, increased ROS formation and a decrease of the mitochondrial membrane potential.^[31]^ Collectively, there are range of V^IV^‐compounds published, which all highlighted their importance in the treatment of different cancers. Importantly, the reports suggest both interactions with DNA and apoptosis by generation of ROS, are the major contributors to cytotoxicity and modes of action.

In 2019, we reported a series of bis(β‐diketonato) oxovanadium(IV) complexes (**1**–**9**) through a facile and straightforward solid state synthesis, and show the complexes exhibit high cytotoxicity and selectivity towards cancer cells, with IC_50_ values up to 11.5× higher than cisplatin.^[32]^ The complexes are also stable under physiological conditions and UV/Vis studies show interaction with BSA (bovine serum albumin). Since compound **9** was previously reported to have increased binding to both BSA, and inserted into the minor groove of the DNA duplex with a partial intercalation,^33]^ we have been interested in understanding the potential modes of action bis(β‐diketonato) oxovanadium(IV) complexes. Herein, we report studies on the compound's cytotoxicity, interactions with DNA (plasmid, genomic and AT‐ and GC‐rich sequences), production of reactive oxygen species (ROS) and modes of cell death by apoptosis and Caspase‐3/7.

## Results and Discussion

### Synthesis and Characterization of bis(β‐diketonato) oxovanadium(IV) complexes

**Scheme 1:**
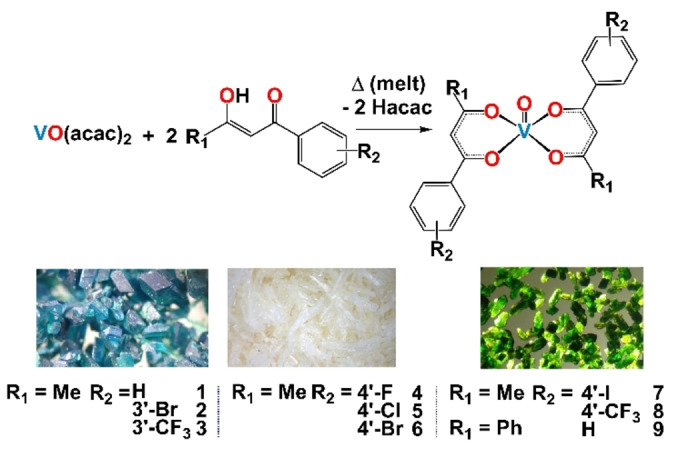
The synthesis of bis(β‐diketonato)vanadyl(IV) compounds **1**–**9**, with an image of the crystals obtained before and after the dry‐melt method.^[30]^

**Figure 1:**
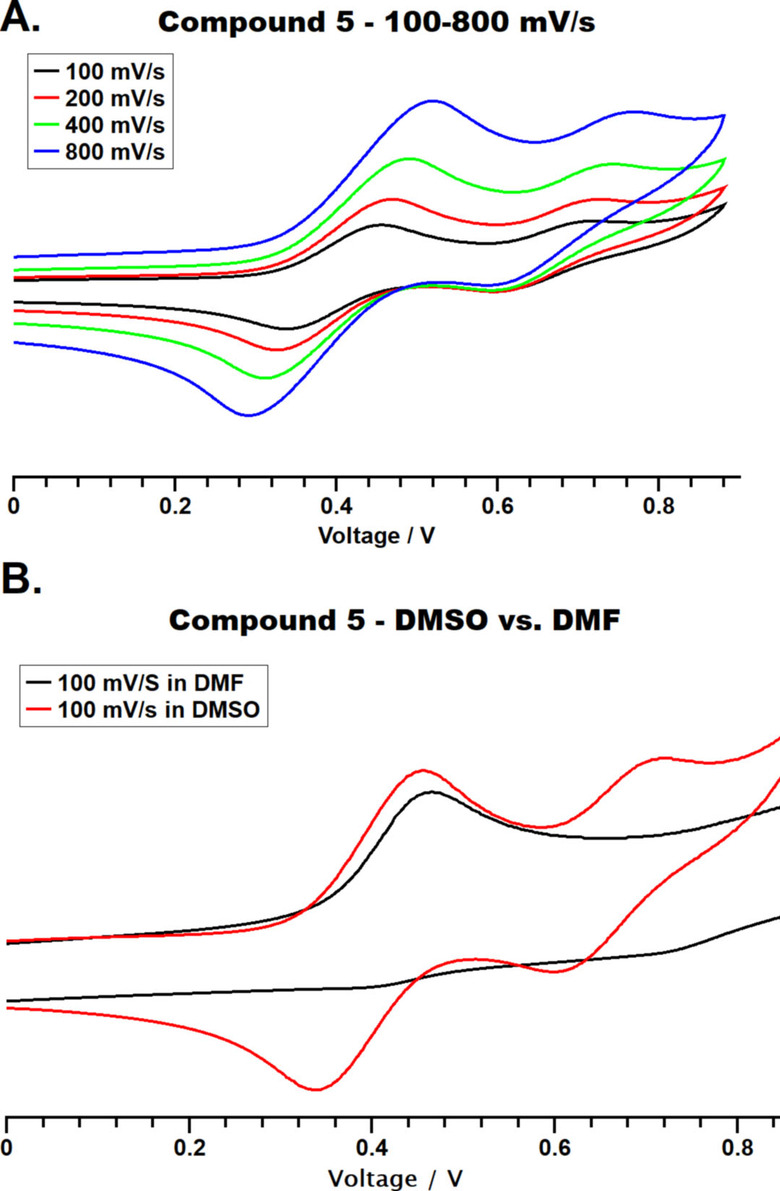
Cyclic voltammograms of compound **5** in A. dry DMSO/0.1 m NBu_4_PF_6_; scan rate=100‐800 mV/s; B. dry DMSO (red) or dry DMF (black)/0.1 m NBu_4_PF_6_; scan rate=100 mV/s. All potentials are reported are referenced against ferrocene (Fc/Fc^+^=0.0 V).

We have recently highlighted the rapid (<5 mins) dry‐melt synthesis and anti‐cancer screening of nine bis(β‐diketonato) oxovanadium(IV) complexes (**1**–**9**, Scheme 1).^[32]^ Alongside other researchers, we have reported that these vanadyl(IV) complexes have the potential to form vanadium(V) in solution. In order to address the compounds stability in DMSO, **1**–**9** and VO(acac)_2_ have been studied by cyclic voltammetry (CV) in dry DMSO under a flow of argon. All compounds (∼10 mm) display *quasi‐*reversible behavior, where the forward and reverse electron transfer reactions occur with different rates. Notably, no CV spectra were obtained for the free ligands. In the presence of all vanadium compounds, there is a one‐electron process for the β‐diketonate ligands (two ligands), which is represented by the oxidation Epa_1_ and reduction Epc_1_. Followed by a weaker *quasi* reversible one electron process for the vanadium V^IV^→V^V^, with oxidation Epa_2_ and reduction Epc_2_ (Table S1). Previous literature has highlighted the irreversible oxidation of VO(acac)_2_ and VO(acac)L (L=aminophosphinic acid) when using 0.1 m NaCl (glass electrode),^[34]^ whilst other reports have noted VO(acac)(abp) (abp=2‐acetylpyridine‐benzoylhydrazone) has a strong abp ligand oxidation at 0.8 V but no observable oxidation for the vanadium (0.1 m PTBA/CH_2_Cl_2_).^[35]^ However, the *quasi‐*reversible nature of our vanadyl complexes is evident when the complexes are scanned at faster scan rates (100–800 mV/s, Figures S1–S13), where the rates of reaction are not sufficiently well match to maintain Nernstian equilibrium and ΔE_p_ increases (e. g. compound **5** in Figure 1A).

**Figure 2:**
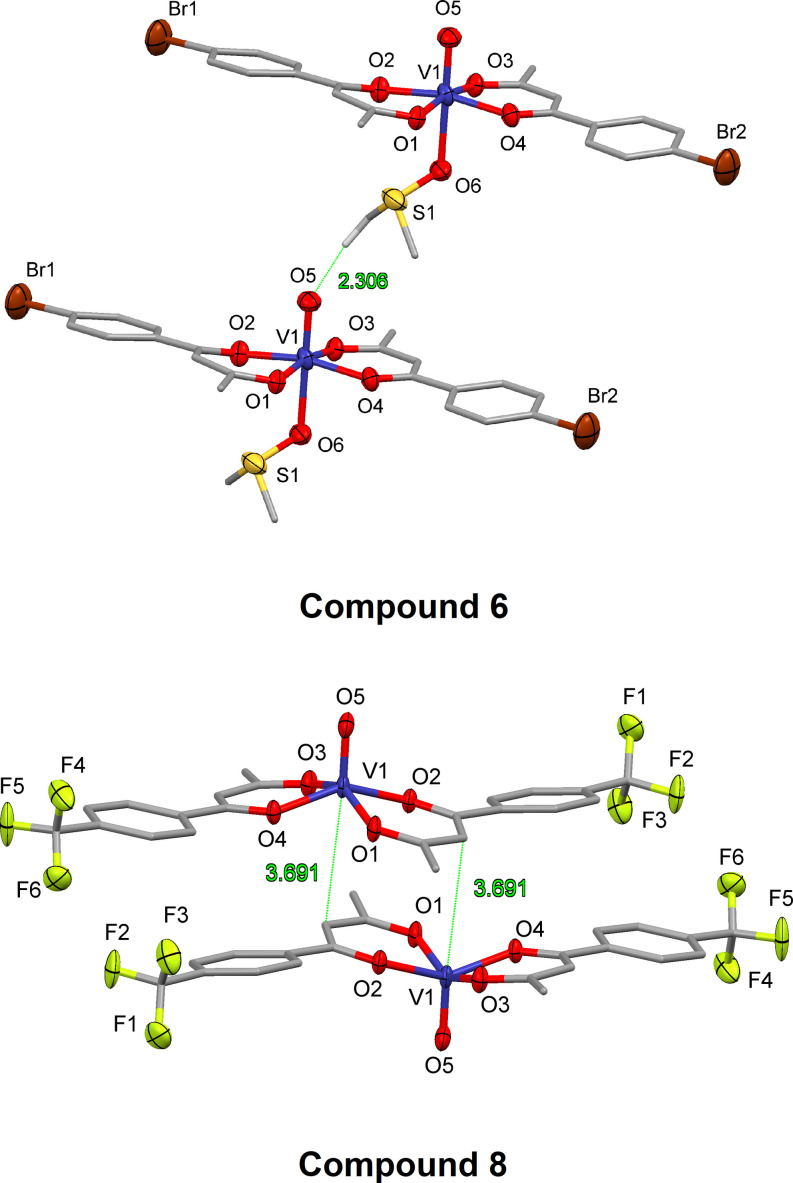
Packing diagram of compound **6** (DMSO‐adduct) and compound **8** (adduct‐free). Showing the interactions of the vanadyl with neighbouring molecules.^[32]^

When the CV measurements were conducted in other dry solvents, either dry dimethylformamide (DMF; compound **5** in Figure 1B) or dry acetonitrile (MeCN, VO(acac)_2_ and compound **3** in Figure S6), the reversibility of the vanadyl(IV) is compromised. These CV measurements show the revisbility is solvent dependent, and this could be due to the interactions of the solvent with the metal center. Although all spectra were conducted using analytically pure vanadium sample from a solvent‐free dry‐melt reaction (Scheme 1); we have previously shown these complexes often crystallize with DMSO solvent in the 6^th^ coordinate position. Figure 2 shows the packing of the complexes, when the 6^th^ coordinate position is occupied by DMSO (**6**) or vacant (**8**) and highlight the interactions between two adjacent molecules. These interactions may have the ability to change the polarity of the V=O bond and alter the redox reversibility.

### Cytotoxicity Studies

The *in vitro* antiproliferation activity of complexes **1**–**9** was evaluated by the MTT assay at dosages between 0.1–100 μm, and repeated to confirm our previous cytotoxicity studies on these complexes.^[32]^ Screening was conducted against of two human cancer cell lines, lung (A549) and pancreatic (MIA PaCa‐2), and the normal retinal epithelium (RPE‐1) cell line. The half maximal effective doses (IC_50_) were calculated after cells were incubated with test complexes between 24 h and 96 h. In most cases, the complexes were either non‐toxic or only moderately cytotoxic between 24–48 h and required longer incubation periods to become cytotoxic, therefore, only data for 72 h and 96 h incubation periods are presented in Table S2. A549 cells were selected for further cell‐based studies due to their lower IC_50_ values and significant reduction in cell viability, whilst compounds **2**, **3**, **8** and **9** were selected for further *in vitro* analysis, due to their varied cytoxicity values across all cell lines and increased sensitivity towards A549 (Table S2).

### Complex‐DNA interaction by agarose gel mobility assay

**Figure 3:**
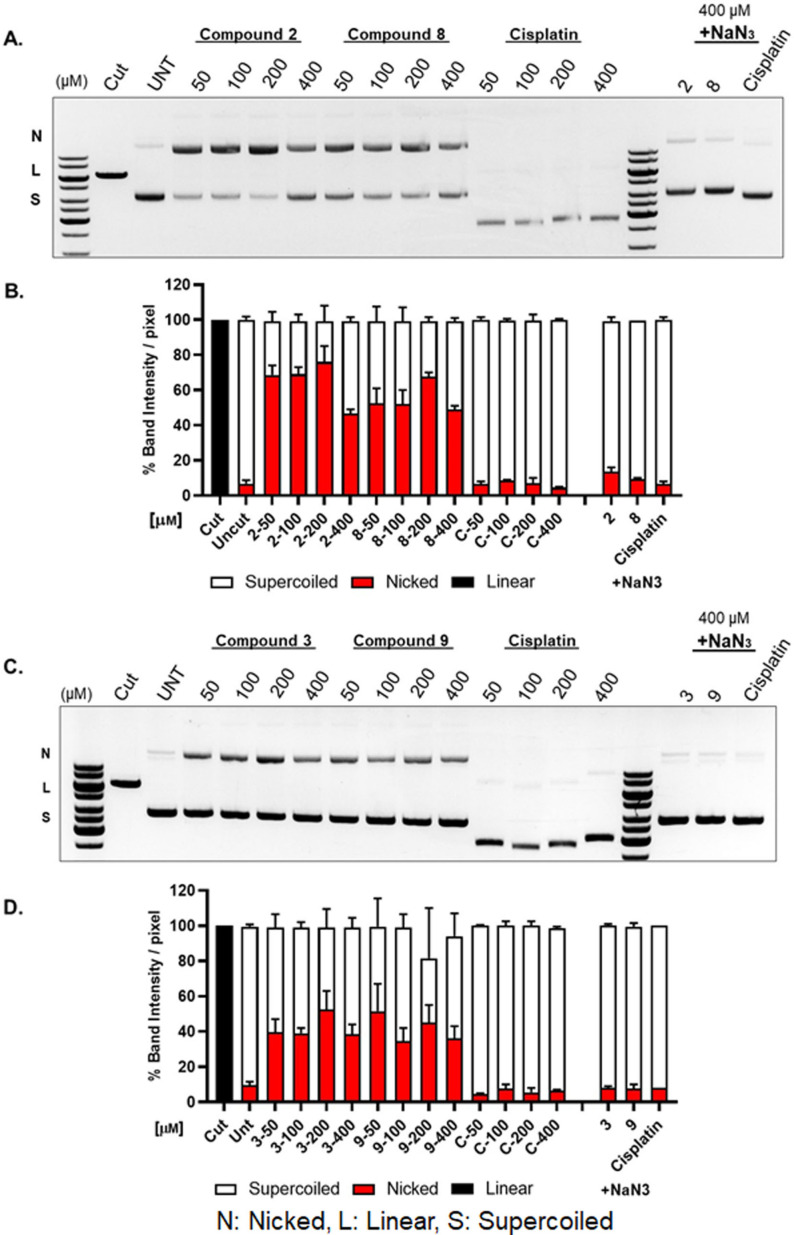
Gel electrophoresis *in vitro* analysis of compounds **2**, **3**, **8** and **9** and plasmid DNA. Plasmid DNA was incubated with different concentrations of compound (50 to 400 μm) for 24 h at room temperature. NaN_3_ was used as a ROS scavenger. The intensity of the DNA bands in each lane was quantified and represented as mean±SD (n=2) for nicked circular, linear and super coiled. A and C: Representative gel images, B and D: Quantification of gels from A and C.

The intercalating, nicking or cleaving capacity of DNA can be determined by gel electrophoresis using plasmid DNA. *In vitro* plasmid DNA assays were conducted by incubating 100 ng/mL plasmid DNA with varying concentrations (6.25–400 μm, lower concentrations are shown in Figure S16) of complexes **2**, **3**, **8** and **9**. The resulting fragments were separated by agarose gel electrophoresis and possible changes in the migration pattern of the DNA were assessed. In accordance with previous reports,^[30]^ cisplatin treatment resulted in faster migration of the plasmid DNA as a result of its DNA‐crosslinking activity, and no effect on DNA nicking. On the other hand, all the tested complexes significantly reduced the supercoiled form and exhibited nicking activity as visualized by the increase in closed circular band and decrease in the supercoiled form (Figure 3). These nicks did not appear to lead to double stranded DNA breaks (DSBs), since no linear band was detectable. As sodium azide (NaN_3_) can be used to scavenger singlet oxygen (^1^O_2_), it can be used to determine if reactive oxygen species (ROS) were involved in these reactions. The addition of NaN_3_ completely rescued and reversed all the effects after incubation with our complexes, even at the highest tested concentration, and this gives strong indication of the involvement of ^1^O_2_ radicals (Figure 3, right lanes).

### Complex‐DNA Interactions

Förster resonance energy transfer (FRET) based DNA melting experiments were used to measure complex‐induced stabilization or destabilization of DNA, by comparing the melting temperature of DNA (0.2 μm) in the absence and presence of our complexes. When the melting temperatures of the DNA (*T*_m_) are higher in the presence of a compound, this indicates a stabilization of the DNA.[^36^, ^37^] The Δ*T*_m_ of double strand (DS) DNA was measured in the presence of 1 μm (5 eq.) of complexes **1**–**9** (Table 1 and Figures S17–S25). Under these conditions, there was no effect on the stability of DNA for any of the complexes examined. As we observe effects on DNA in our other experiments at a higher concentration of complex, we also performed the FRET melting at 25 μM (Table S3 and Figures S26–S30), consistent with our DNA fragmentation assay. Upon increasing the concentrations of complex **2**, **3**, **8** and **9** to 25 μm, there was no significant change in melting temperature of the DNA (Table 1). This indicates that the complexes do not stabilize or destabilize the DNA under these conditions. Given the results observed using gel electrophoresis we were interested in the relative binding affinity of the complexes to DS DNA. We performed a fluorescence intercalator displacement (FID) assay to determine the displacement of thiazole orange (TO) in the presence of the complexes.[^38^, ^39^]

**Table 1:** FRET melting Δ*T*_m_ with DS DNA (0.2 μm) and compounds **1**–**9** (1 μm or 25 μm). FID % TO displacement of 5 μm or 25 μm complex with 1 μm of a DNA buffer: 100 mm potassium chloride and 10 mm sodium cacodylate at pH 7.4. Errors represent the standard deviation from triplicate repeats.

	FRET Melting Δ*T*_m_ [°C]		TO Displacement *D*_TO_ [%]					
	DS		DS		AT6		GC6	
μM	1	25	5	25	1	25	1	25
**1**	0.0±0.3	–	3±2	–	7±3	–	2±1	–
**2**	0.0±0.3	0.3±0.2	9±2	59±3	26±4	79±9	20±5	71±4
**3**	−0.2±0.7	0.7±1.0	10±2	15±2	22±4	34±4	17±4	20±2
**4**	−0.8±0.4	–	1±2	–	8±3	–	6±1	–
**5**	−0.6±0.2	–	5±5	–	24±2	–	15±3	–
**6**	−0.3±0.3	–	9±9	–	12±1	–	12±1	–
**7**	−0.3±0.3	–	13±2	–	32±1	–	16±2	–
**8**	−0.4±0.2	0.4±0.3	16±2	17±2	30±2	39±4	21±4	22±3
**9**	−0.3±0.3	0.0±0.2	27±4	36±3	47±3	66±5	29±2	42±2

**Figure 4:**
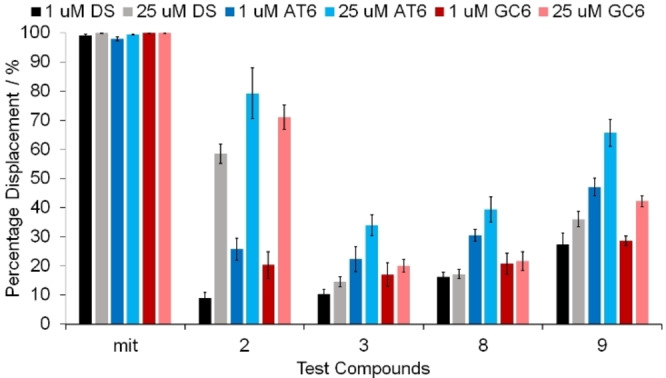
Thiazole orange (TO) displacements (%) for 1 μM (black=DS, dark blue=(AT)_6_ and dark red=(GC)_6_) and 25 μM (grey=DS, light blue=(AT)_6_ and light red=(GC)_6_) with test compounds: Mitoxantrone (mit, positive control) and compounds **2**, **3**, **8** and **9**. Error bars represent the standard deviation from triplicate repeats.

We performed the displacement assay in the presence of DS DNA, as well as sequences comprised of (AT)_6_ and (GC)_6_. This would reveal not only the affinity for DS DNA but assess whether there was any preference for AT‐ or GC‐rich DNA regions. There was a range of relative binding affinities for the complexes (Table 1). Complexes **1** and **4** had very low relative binding, indicated by low displacement of TO from the DNA. The remainder of the complexes had moderate to high affinity for the DNA. Complex **9** showed the best displacement of DS DNA (27 %) at 5 μm complex concentration (5 eq.). Additionally, complexes **5**, **7**, **8** and **9** showed a higher displacement for (AT)_6_ compared to (GC)_6_. Out of these complex **9** was found to have the strongest relative binding for (AT)_6_ with a TO displacement of 47 % at 1 μm of complex. However, complex **7** has the highest specificity for (AT)_6_ over (GC)_6_ ((AT)_6_=32 % vs. (GC)_6_=16 %). In line with our other experiments, additional studies were conducted with complexes **2**, **3**, **8** and **9** at the higher concentration of 25 μm (Figure 4), which showed further displacement of TO for complexes **2** and **9**. The relative binding of these complexes is consistent with the results observed in the DNA fragmentation assays. Taken together with the FRET melting data, we can conclude that these complexes bind but do not stabilize DS DNA, and this will contribute to the biological effects observed. Complexes **3**, **8** and **9** all show a preference for AT‐rich regions of DNA, which may affect the regions of genomic DNA that they target.

### Reactive Oxygen Species (ROS)

**Figure 5:**
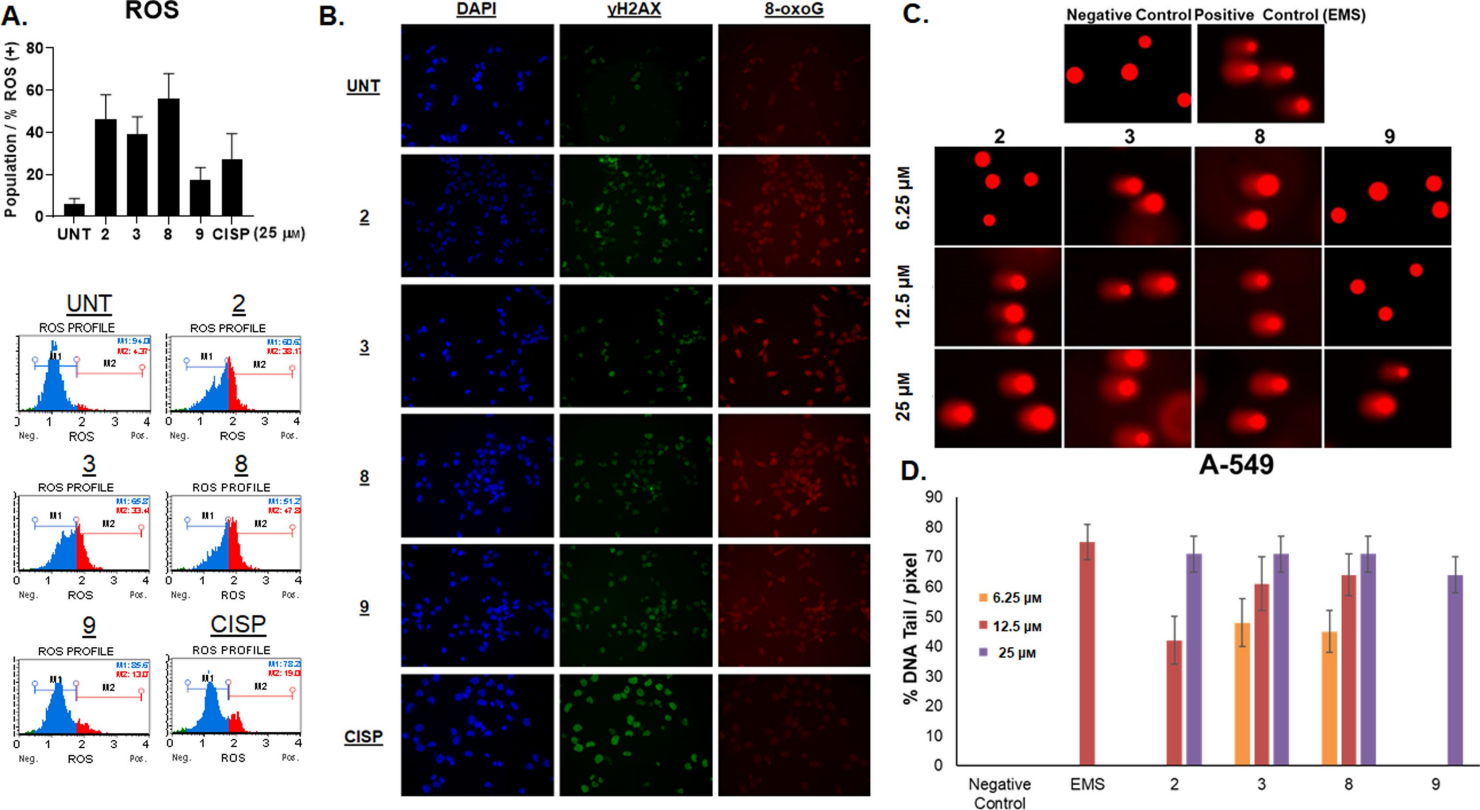
A. Top panel: Increase in the reactive oxygen species (ROS) upon treatment with **2**, **3**, **8**,**9** and cisplatin (25 μm, 48 h). Bottom panel: Representative flow profiles ‐ M1 indicates cell population without ROS (ROS−) while M2 is cell population with ROS (ROS+). B. Microscopic visualization of DNA damage exerted by **2**, **3**, **8**, **9** and cisplatin. DNA was visualized through DAPI staining. 8‐oxoG staining was used to determine oxidative DNA lesions, and ɣH2AX to determine DSBs. C. Images of nuclei from the COMET assay, showing quantitative genomic damage. Representative images of nuclei following treatment with **2**, **3**, **8** and **9** . D. Quantification of tail formation from the COMET assay.

As it is well documented that singlet ^1^O_2_ species cause cell death, we hypothesized that the complexes may elevate intracellular ROS in cells, leading to loss of cell viability. To test that, cellular ROS levels were monitored using dihydroethidium, a well described reagent to detect oxidative stress in cell populations. Indeed, all complexes resulted in an increase in ROS, albeit with varying degrees (13–48 % as opposed to 4 % in untreated controls). In comparison, cisplatin induced ROS was on the lower end with a mild 19 % increase (Figure 5A). The flow charts represent the distribution of two populations, M1 (blue) peaks indicate cell population without ROS (ROS−), while M2 (red) is cell population with ROS (ROS+). To further expand on the DNA damage induced in cells in response to our complexes, A549 cells (visualized by DAPI staining, Figure 5B, left panel) were stained for the presence of 8‐oxo‐Guanidine, which is the most common and abundant type of lesion exerted on the DNA in response to oxidation. As expected, oxidative DNA damage was elevated parallel to the increase in intracellular ROS (Figure 5B, middle panel). Interestingly, cells were also positive for ɣH2AX (Figure 5B, right panel), which is a widely used marker for DSBs, indicating that the complexes can trigger breaks in the cellular environment.

### COMET assay

To further evaluate the genomic damage exerted by complexes **2**, **3**, **8** and **9**, a COMET assay was performed. A549 cells were treated for 48 h with 6.25, 12.5 and 25 μm of the test compounds, which lead to a dose‐dependent increase in tail formation (Figure 5C). The damage was most pronounced at 25 μm which was comparable to the positive control (EMS at 12.5 μm, Figure 5D, top panel). Therefore, all compounds induced oxidative DNA stress, DSBs and genomic damage to considerable extends, possibly as a result of increased ROS induced by our compounds.

### DNA fragmentation and condensation

**Figure 6:**
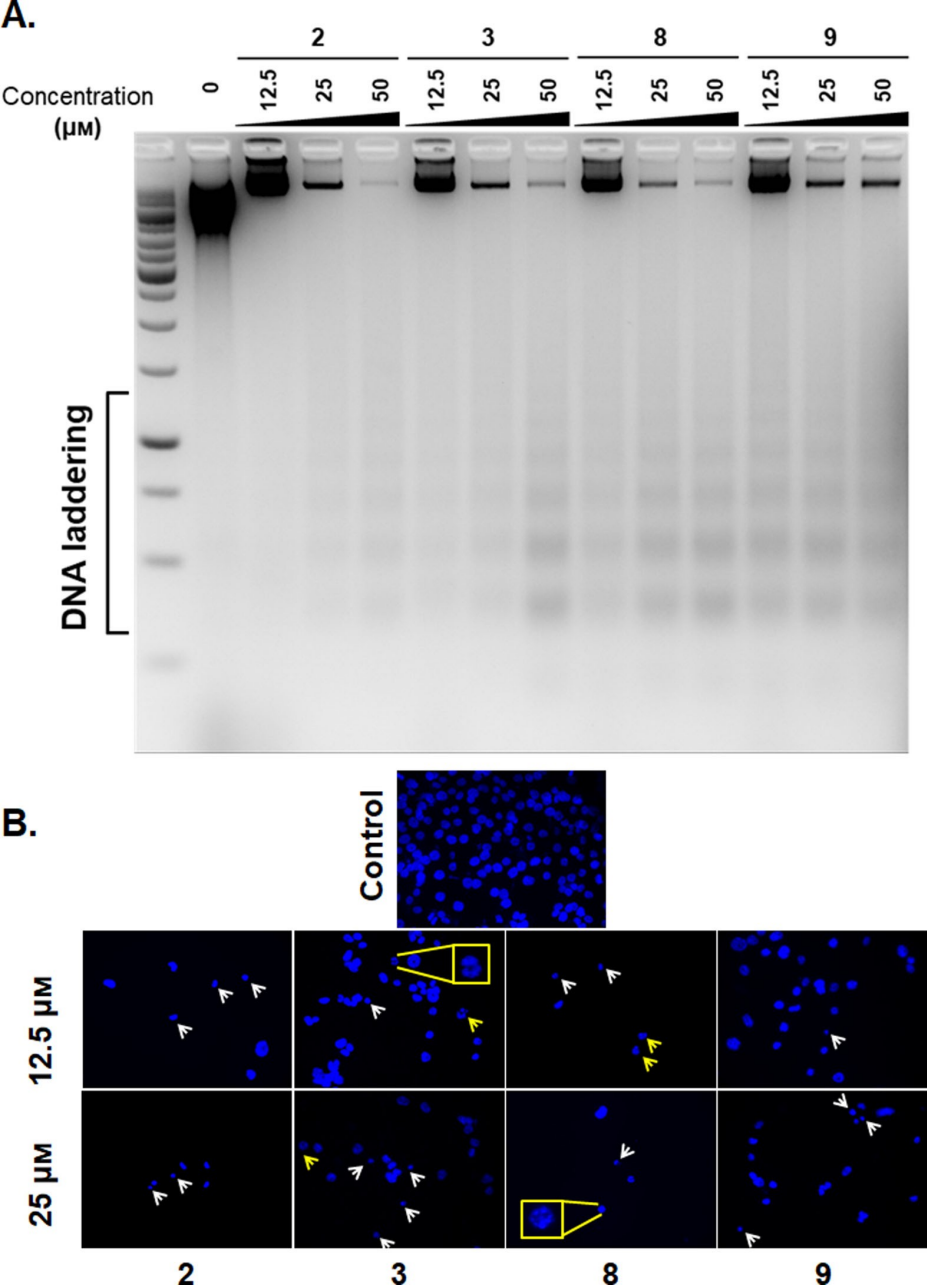
A. DNA fragmentation assay: A549 cells were exposed to indicated doses of **2**, **3**, **8** and **9**, and the genomic DNA was run on agarose gel. DNA laddering occurred in a dose dependent manner. B. Changes in DNA morphology: DNA shrinkage and fragmentation. Cells were exposed to 12.5 and 25 μm of **2**, **3**, **8** and **9** for 72 h and fixed. White arrows indicate DNA shrinkage, yellow arrows indicate DNA fragmentation. Insets show enlarged view of fragmented nuclei.

In order to determine whether our complexes induce programmed cell death pathways, A549 cells were exposed to 12.5–50 μm of complexes **2**, **3**, **8** and **9** for 72 h and genomic DNA was collected. Indeed, each complex induced DNA ladder formation in a dose dependent manner, indicative of DNA fragmentation, which is a hallmark of apoptosis (Figure 6A). To further evaluate this result morphologically, cells were visualized under the microscope for presence of DNA shrinkage and fragmentation, and once again there were several examples of cells exhibiting these changes, supporting apoptosis as the mode of cell death (Figure 6B).

### Apoptosis

**Figure 7:**
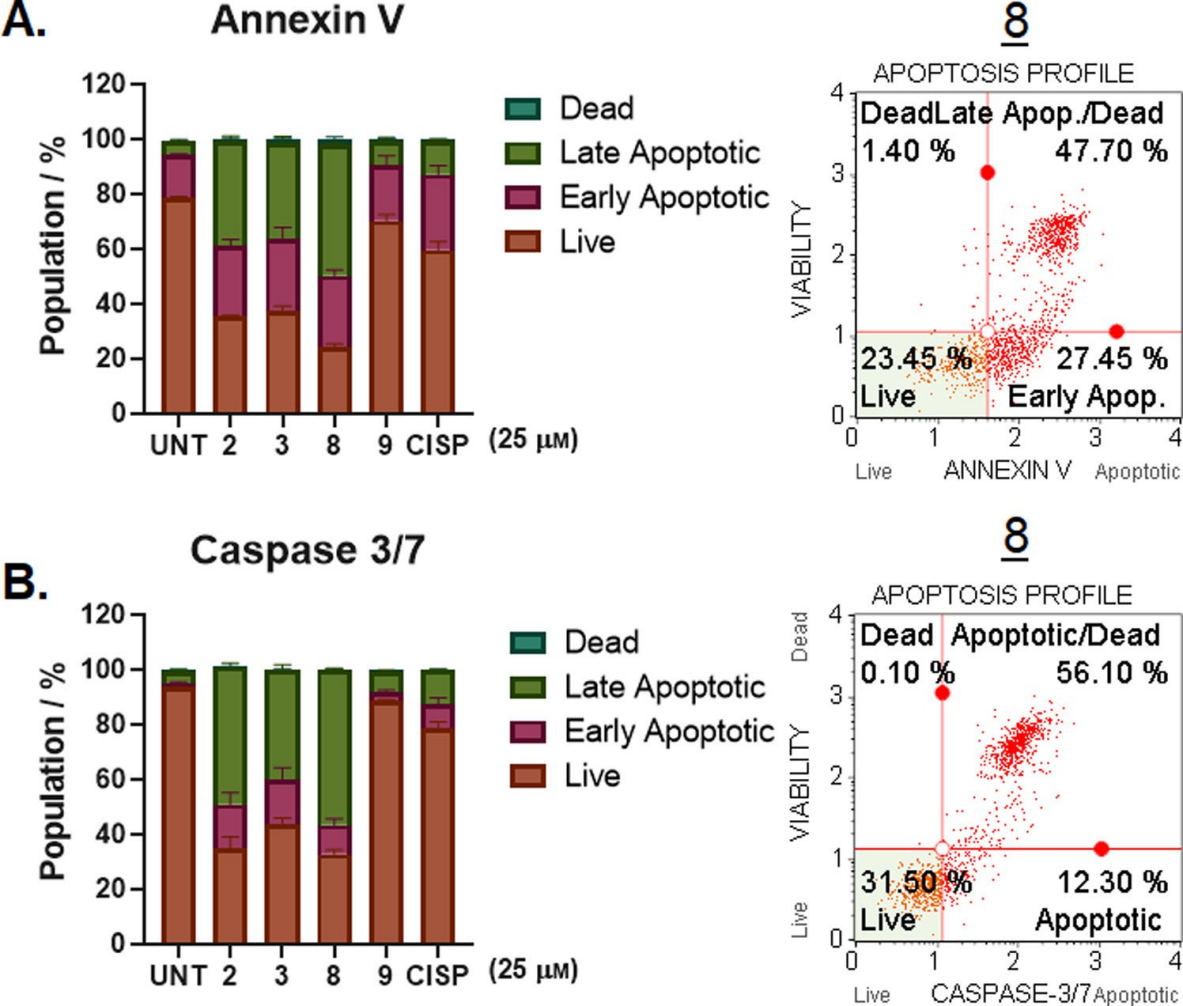
A. Flow cytometric analysis using Annexin‐V staining with 7‐AAD incorporation; B. Flow cytometric analysis of the activated Caspase‐3/7 and simultaneous detection of dead cells by 7‐AAD staining. In both cases, A549 cells were exposed to compounds **2**, **3**, **8** and **9** (25 μm) for 48 h. Percentages of cells in quadrant are shown in the right panel for compound **8** only, and given as non‐apoptotic live (lower left), non‐apoptotic dead (upper left), early apoptotic (lower right), and late‐apoptotic (upper right).

In order to more quantitatively analyze apoptosis, an Annexin‐V assay was used, which is a commonmethod to quantify the number of cells undergoing apoptosis, where flow cytometry is used to separate the cells depending on their uptake of certain dyes. It has previously been reported that when using an Annexin‐V assay to assess apoptosis of V^IV^ complexes in A549 and HeLa cell lines, ∼2 % early stage apoptosis and ∼18 % late stage apoptosis was observed.^[30]^ When compared to the control, all complexes cause an increase in both early and late apoptosis, however, complex **8** has the highest degree of late apoptosis, which was ∼4× greater than cisplatin and 2× greater than recently published V^IV^ complexes (Figure 7A).^[30]^ All complexes exhibit moderate early apoptosis, ranging from 23 % (**9**) to 29 % (**3**), which are comparable with cisplatin (30 %), see Figure S31.

As the executioner Caspases (Caspase‐3/7) play a role in the cleaving of proteins, which leads to apoptotic breakdown, the activity of Caspase‐3/7 was determined after A549 cells were exposed to 25 μm of complexes **2**, **3**, **8** and **9**. Consistent with previous results,^[30]^ Caspase‐3/7 activity was drastically elevated in response to treatment with the complexes compared to the mock treated controls (5.8 %) (Figure 7B). Gratifyingly, while cisplatin led to only a mild increase (∼22 %) in apoptosis, complexes **2**, **3** and **8** induced ∼2–3× higher levels (68 %, 58 % and 68 % respectively) and a slight increase by **9** (10 %), possibly explaining the higher IC_50_ of these compounds (Figure S31). In summary, our cumulative evidence suggested apoptosis as the main form of cell death in response to complexes **2**, **3**, **8** and **9**, and their structure design warrants further investigation for the treatment of lung carcinomas.

## Conclusion

We have reported herein, a study of the modes of action of bis(β‐diketonato) oxovanadium(IV) complexes (**1**–**9**). Cytotoxicity studies were conducted against several cell lines, and highlight the human lung carcinoma cell line, A549 was sensitive to these compounds. Gel electrophoresis using plasmid DNA was conducted, and the tested complexes exhibit significant reductions in the supercoiled form and display DNA nicking activity. However, these nicks do not suggest double stranded DNA breaks (DSBs). The addition of NaN_3_ (single oxygen scavenger), completely rescued and reversed all the effects after incubation with our compounds and gives a strong indication for the involvement of ^1^O_2_ radicals.

Förster resonance energy transfer (FRET) based melting experiments show no significant changes in the DS DNA after incubation with 25 μm (125 eq.) of these bis(β‐diketonato) oxovanadium(IV) complexes. Since the gel electrophoresis results highlighted DNA interactions, the potential binding affinities of the complexes to DS DNA were measured using a fluorescence intercalator displacement (FID) assay. At 5 μm complex concentrations (5 eq.), complex **9** shows the best displacement of DS DNA (27 %), whilst an increase to 25 μm highlighted complex **2** with the best displacement (59 %). Studies were conducted to assess the complexes preferentially binding to either AT‐ or GC‐rich DNA, and complexes **3**, **8** and **9** all show a preference for AT‐rich regions of DNA.

A549 cells were incubated with complexes **2**, **3**, **8** and **9**, and the presence of 8‐oxo‐Guanidine was measured,showing oxidative DNA damage was elevated parallel to the increase in intracellular ROS. Interestingly, cells were also positive for ɣH2AX, indicating that the complexes can trigger DNA breaks in the cellular environment. These compounds were also shown to exhibit dose‐dependent increase in tail formation in genomic DNA when using the COMET assay, showing increases in DS DNA damage. This induction in oxidative DNA stress, DSBs and genomic damage, is possibly as a result of increased ROS induction. Finally, we have shown these compound have induced DNA ladder formation in a dose dependent manner and DNA shrinkage and fragmentation, which all support the Annexin‐V and Caspase‐3/7 results, and highlight increased early and late apoptosis and elevation of Caspase‐3/7; which are all suggestive that apoptosis is the main form of cell death in A549 cells treated with these bis(β‐diketonato) oxovanadium(IV) complexes.

## Experimental Section

General: All ligands and complexes were synthesized under aerobic conditions. All chemicals were supplied by Sigma‐Aldrich Chemical Co. Deuterated NMR solvents were purchased from Sigma‐Aldrich Chemical Co. or Acros Organics. All ligands and complexes were prepared using a previously reported literature methods.^[32]^

Cyclic Voltammetry: Cyclic voltammetric measurements were carried out using Autolab PGStat 30 potentiostat/galvanostat. A single‐compartment or a conventional three‐electrode cell was used with a silver/silver chloride reference electrode (3 m NaCl, saturated AgCl), glassy carbon working electrode and Pt wire auxiliary electrode. Dimethylsulfoxide, DMSO or dimethylformamide, DMF was stored and used over molecular sieves, whilst acetonitrile (MeCN) was freshly distilled from CaH_2_. Tetrabutylammonium hexafluorophosphate [N(C_4_H_9_‐*n*)_4_][PF_6_] was used as the supporting electrolyte. Solutions containing ca. 10 mm analyte (0.1 m electrolyte) were degassed by purging with argon, and spectra were collected with a constant flow of argon. All spectra were referenced to ferrocene/ferrocenium.

Cell Culture: A549 (ATCC, CCL‐185), MIA PaCa‐2 (ATCC, CRL‐1420), and RPE‐1 cells (ATCC, CRL‐4000)) were all grown in Dulbecco's Modified Eagle Medium F‐12 (Gibco #11320033) containing 10 % FBS (Gibco, 10500064) and Penicillin‐Streptomycin (10,000 U/mL) (Gibco, 15140122) in a 37 °C, 5 % CO_2_ incubator.

Cell viability: Stock solutions of all complexes were prepared in DMSO (10 mm) and aliquots were stored at −20 °C. 8×10^3^ (MIA PaCa‐2), 4×10^3^ (A549) and 2×10^3^ (RPE‐1) cells were seeded on 96‐well plates and incubated with serial dilutions of the complexes (freshly prepared, 0–100 μm) for 72 and 96 h (or 24 and 48 h, see *Supplementary Information*). Cell viability was measured via MTT assay. Briefly, 20 μL of MTT reagent (Sigma Aldrich, M‐5655) was added on the cells (5 μg/mL, at 37 °C). After 4 h, formazan crystals were solubilized in 10 % SDS, 0.01 m HCl (50 μL, overnight at 37 °C), and color formation was detected with Bio‐Tek H1 Synergy microplate reader.

Agarose gel electrophoresis for complex/DNA interactions: The complex‐DNA interactions were evaluated *in vitro* as described previously with minor changes.^[40]^ Briefly, plasmid DNA (200 ng, pBOS‐H2BGFP, BD Biosciences) was incubated with **2**, **3**, **8**, **9** or cisplatin for 24 h (50–400 μm, in a total reaction volume of 20 μL, (RT). NaN_3_ (37.5 mm in ddH_2_O, final concentration) was used for ROS scavenging activity. Plasmid DNA was linearized via digestion with BamHI (Thermo Scientific, ER0051), and samples were run on 1 % agarose gel (100 V, 60 min). The experiment was performed with two biological repeats.

ɣH2AX and 8‐oxoG staining: A549 cells were treated with 25 μm of **2**, **3**, **8**, **9** and cisplatin for 24 h and fixed in freshly prepared 4 % paraformaldehyde (15 min at room temperature (RT)), permeabilized in 0.1 % Triton X‐100/PBS (1 h, RT), blocked in 0.2 % gelatin (1 h, RT) and stained with ɣH2AX (Cell Signaling, #9718S, 1 : 400) or 8‐oxo‐Guanine (EMD Millipore, MAB3560, 1 : 100) antibodies overnight at 4 °C. Next day, cells were stained with Goat Anti‐Rabbit IgG H&L (Alexa Fluor® 488) (ab150077) and Goat Anti‐Mouse IgG H&L (Alexa Fluor® 594) secondary antibodies for 1 h, at RT. The cover slips were mounted on slides with VECTASHIELD Antifade Mounting Medium with DAPI (Vector Laboratories, H‐1200) and imaged with Zeiss Axio Imaging M1 fluorescence microscopy under 40x magnification.

Detection of Reactive Oxygen Species: 2×10^5^ A549 cells were seeded onto 6‐well plates and treated with 25 μ m of **2**, **3**, **8** and **9** for 48 h. Cells were trypsinized and centrifuged (300 g, 5 min). The pellet was resuspended with 1×Assay Buffer (MCH100111‐2). Oxidative Stress Reagent (4700‐1665) was diluted with 1×Assay Buffer in a 1 : 100 ratio to prepare intermediate solution. This solution was further diluted with 1×Assay Buffer in 1 : 80 ratio to prepare a working solution. 50 μL cell suspension was mixed with 150 μL working solution and incubated at 37 °C for 30 min. ROS activated cells were counted with Muse Cell Analyzer (Merck Millipore).

COMET assay: Genotoxicity of the complexes was evaluated using previously published protocols^[41]^ with slight modifications. Briefly, 4×10^4^ A549 cells were seeded on 24‐well plates and incubated for 24 h (37 °C, 5 % CO_2_). Serial dilutions of **2**, **3**, **8**, or **9** (freshly prepared at 6.25, 12.5, and 25 μm concentrations) were added on the cells and incubated for 48 h. DMSO (Sigma, D2650) was used as negative control, while ‘Ethyl methanesulfonate’ (EMS – Merck‐Millipore #8.20774) served as positive control (40 mm, 1 h) under the same experimental conditions. At the end of incubation, cells were counted using a haemocytometer, and the resuspended in PBS to a final concentration of 1.6×10^4^ cells/mL. Cells were then mixed with low melting agarose, spread over the slides, and subjected to lysis buffer (at dark, +4 °C, 24 h). The slides were washed with an alkaline carrier buffer (pH≥13, 20 min), electrophoresed (13 V, 0.03 mA, 25 min), and stained with propidium iodide (10 μg/mL, #P4864 Sigma‐Aldrich, 20 min). They were visualized by a fluorescence microscope (535 nm/617 nm wavelength, 40X magnification) and quantified using ImageJ software. All experiments were done in two biological repeats, and 50 individual cells/experiment were quantified.

Annexin‐V staining and determination of Caspase‐3/7 activity: 2×10^5^ A549 cells were seeded onto 6‐well plates and treated with 25 μm of **2**, **3**, **8** and **9** for 48 h. Cells were washed in PBS, trypsinized and analyzed using Muse Cell analyzer (Millipore). Annexin V/Dead Cell (Luminex, MCH100105) and Caspase‐3/7 (Luminex, MCH100108) staining were performed using following manufacturer's instructions with the changes as previous reported.^[29]^

DNA Fragmentation assay: A modified protocol^[40]^ was used for DNA fragmentation assay. Briefly, 2.5×10^5^ cells were seeded onto 25 cm^3^ flasks and treated with **2**, **3**, **8**, **9** at indicated doses for 72 h. Both floating and attached cells were collected and combined. The cells were washed with PBS and centrifuged (1000 g, 5 min, RT). The pellet was dissolved in 120 μL of lysis buffer [10 mmol/L Tris (pH 7.4), 100 nmol/L NaCl, 25 mmol/L EDTA, 1 % N‐lauryl sarcosine, and proteinase K (final concentration: 0.35 mg/mL)] by gently vortexing, and was incubated at 45 °C for 2 h. The lysates were further incubated for 1 h at RT following addition of 2 μL of RNAse A (10 mg/mL). The samples were resolved on 2 % agarose gels (stained with Ethidium Bromide, 60 V, 5 h) and analyzed using a Bio‐Rad Gel Imaging System.

Oligonucleotides: Oligonucleotides were purchased from Eurogentec and purified using reverse phase HPLC. The dry DNA was dissolved in ultrapure (100 μm for labelled oligos, 1 mm for unlabelled oligos) final concentrations were determined using a NanoDrop; further dilutions were carried out in 10 mm sodium cacodylate supplemented with 100 mm potassium chloride, pH 7.4. Samples were thermally annealed in a heat block at 95 °C for 5 min and cooled slowly to RT overnight.

FRET Based DNA Melting Experiments: Assessment of the ligand‐induced change in melting temperature was performed using a fluorescence resonance energy transfer (FRET) DNA melting based assay.^[34]^ The labelled oligonucleotide with a donor fluorophore FAM (6‐carboxyfluorescein) and acceptor fluorophore TAMRA (6‐carboxytetramethyl‐rhodamine). DS_FRET_ FAM‐d(TAT‐AGC‐TAT‐A‐HEG(18)‐TAT‐AGC‐TAT‐A)‐TAMRA‐3′). Strip‐tubes (QIAgen) were prepared by combining 20 μL of 0.2 μm DNA with the respective complex. Control samples for each run were prepared with the same quantity of solvent with the DNA in buffer. Fluorescence melting curves were determined in a QIAgen Rotor‐Gene Q‐series PCR machine, using a total volume of 20 μL. Samples were held at 25 °C for 5 min then ramped to 95 °C, at increments of 1 °C, holding the temperature at each step for 1 min. Measurements were made with excitation at 470 nm and detection at 510 nm. Final analysis of the data was carried out using QIAgen Rotor‐Gene Q‐series software and Origin or Excel. *T*_m_ values were determined using the first derivative of the melting curves.

Fluorescence Intercalator Displacement (FID): The FID assay was performed on a BMG CLARIOstar plate reader using an excitation of 430 nm and emission was measured from 450 to 650 nm with the emission at 450 nm being normalised to 0 %. 96‐well plates (Corning 96 well solid black flat bottom plates) were used for this assay. 90 μL of thiazole orange (TO) at a concentration of 2 μL in 10 mm sodium cacodylate and 100 mm potassium chloride that was pH corrected to pH 7.4) was added to each well. The fluorescence was then measured at 450 nm with an excitation of 430 nm and normalised to 0 %. 1 μm of DNA was added, shaken at 700 rpm in the plate reader for 30 s and left to equilibrate for 20 min. After equilibration the fluorescence was measured again and normalised to 100 %. After that, additions in to each well (in triplicate) the complexes were added at the stated concentrations. The fluorescence was measured after each addition and normalised between the 0 and 100 % levels previously determined. The percentage displacement of TO value (*D*_TO_) was calculated from the displacement of TO after the addition of complex. DS=5′‐d[GGC‐ATA‐GTG‐CGT‐GGG‐CGT‐TAG‐C]‐3′+complementary sequence. (AT)_6_=5′‐d[ATATATATATAT]‐3′. (GC)_6_=5′‐d[GCGCGCGCGCGC]‐3′ The sequences (AT)_6_ and (GC)_6_ are self‐complementary so were annealed at 2 μm to give 1 μm of duplex overall.

## Conflict of interest

The authors declare no conflict of interest.

## Supporting information

As a service to our authors and readers, this journal provides supporting information supplied by the authors. Such materials are peer reviewed and may be re‐organized for online delivery, but are not copy‐edited or typeset. Technical support issues arising from supporting information (other than missing files) should be addressed to the authors.

Supporting Information
