# Supplementary material for: Understanding the Potential In Vitro Modes of Action of Bis(β‐diketonato) Oxovanadium(IV) Complexes
Source: ChemMedChem. 2021 May 26;16(15):2402–10. doi: 10.1002/cmdc.202100152 (PMC8453837; doi:10.1002/cmdc.202100152)
Supplement: Supplementary file 1 — Supporting Information [file CMDC-16-2402-s001.pdf]

ChemMedChem

Supporting Information

# Understanding the Potential *In Vitro* Modes of Action of Bis (β-diketonato) Oxovanadium(IV) Complexes

Baris Sergi[+], Ipek Bulut[+], Ying Xia, Zoë A. E. Waller, Yasemin Yildizhan, Ceyda Acilan,* and Rianne M. Lord*

# Contents

Cyclic Voltammograms ........ 2

Cytotoxicity Studies ........ 7

$IC_{50}$ values ........ 7

Microscope Images ........ 8

Complex–DNA interaction by agarose gel mobility assay. ........ 10

FRET and FID Biophysical Measurements ........ 11

Raw Data for Compounds 1-9 at 1 μM (5 eq.) ........ 11

Compound 1 ........ 11

Compound 2 ........ 11

Compound 3 ........ 12

Compound 4 ........ 12

Compound 5 ........ 13

Compound 6 ........ 13

Compound 7 ........ 14

Compound 8 ........ 14

Compound 9 ........ 15

Results with 25 μM of test compound ........ 15

Raw Data for Compounds 2, 3, 8 and 9 at 25 μM (125 eq.) ........ 16

Compound 2 ........ 16

Compound 3 ........ 16

Compound 8 ........ 17

Compound 9 ........ 17

Apoptosis and Caspase-3/7 ........ 18

## Cyclic Voltammograms

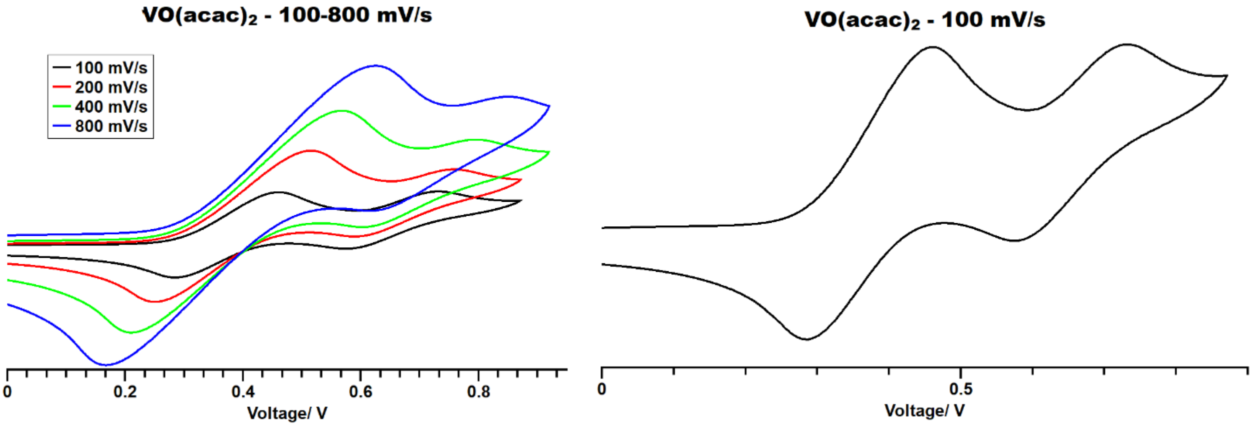


*Figure S 1. Cyclic voltammograms of $VO(acac)_2$ in dry DMSO/ 0.1 M $NBu_4PF_6$. Potentials are reported against ferrocene (Fc/Fc+ = 0.0 V).*

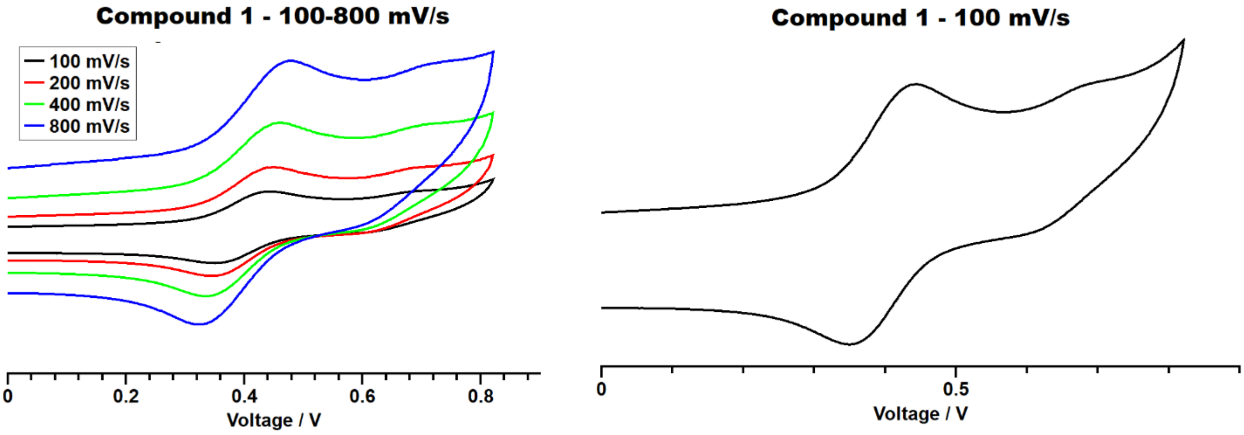


*Figure S 2. Cyclic voltammograms of compound* ***1*** *in dry DMSO/ 0.1 M $NBu_4PF_6$. Potentials are reported against ferrocene (Fc/Fc+ = 0.0 V).*

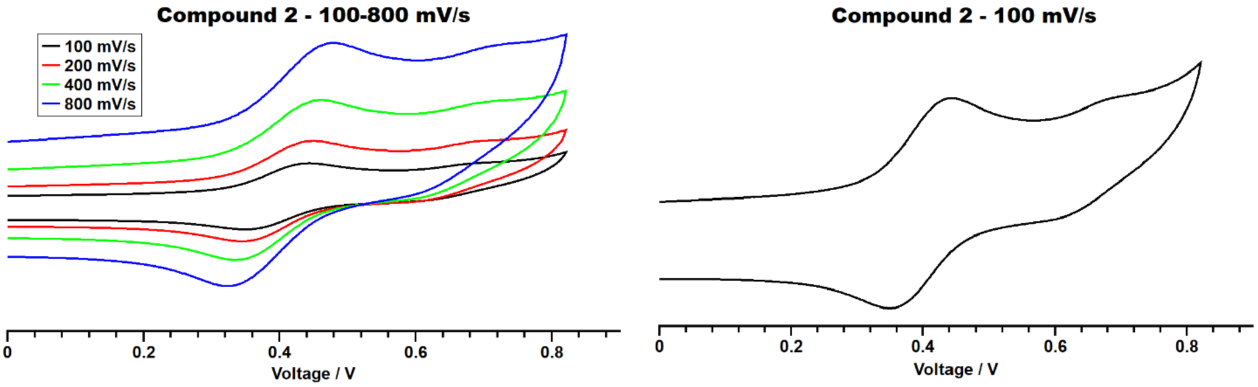


*Figure S 3. Cyclic voltammograms of compound 2 in dry DMSO/ 0.1 M $NBu_4PF_6$. Potentials are reported against ferrocene (Fc/Fc+ = 0.0 V).*

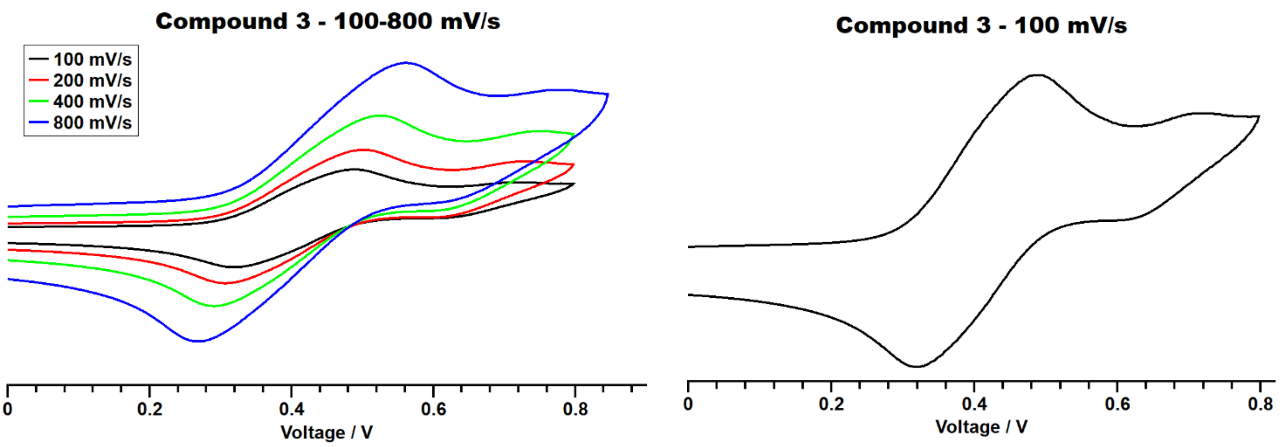


*Figure S 4. Cyclic voltammograms of compound **3** in dry DMSO/ 0.1 M $NBu_4PF_6$. Potentials are reported against ferrocene (Fc/Fc+ = 0.0 V).*

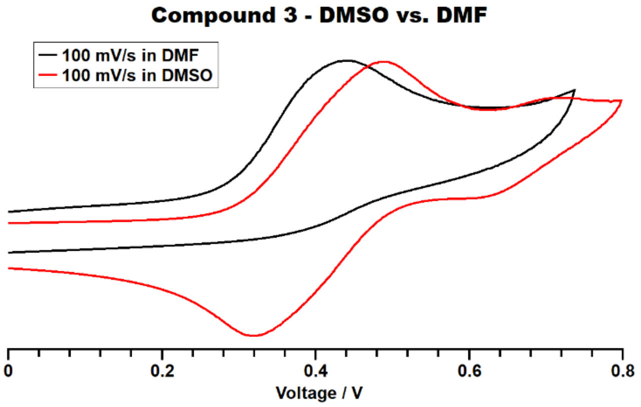


*Figure S 5. Cyclic voltammograms of compound **3** in either dry DMSO (red) or dry DMF (black)/ 0.1 M $NBu_4PF_6$. Potentials are reported against ferrocene (Fc/Fc+ = 0.0 V).*

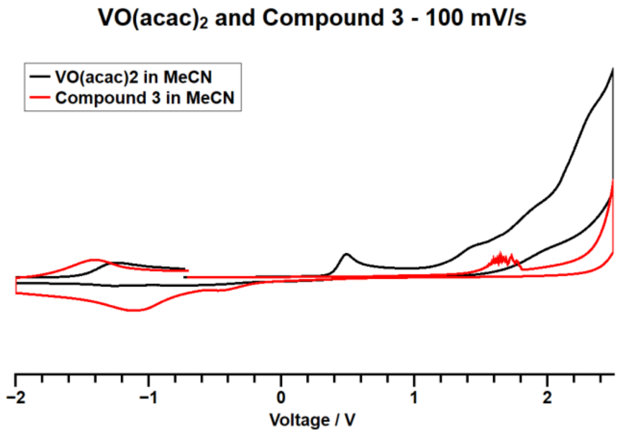


*Figure S 6 Cyclic voltammograms of $VO(acac)_2$ (red) and compound **3** (black) in dry MeCN/ 0.1 M $NBu_4PF_6$. Potentials are reported against ferrocene (Fc/Fc+ = 0.0 V).*

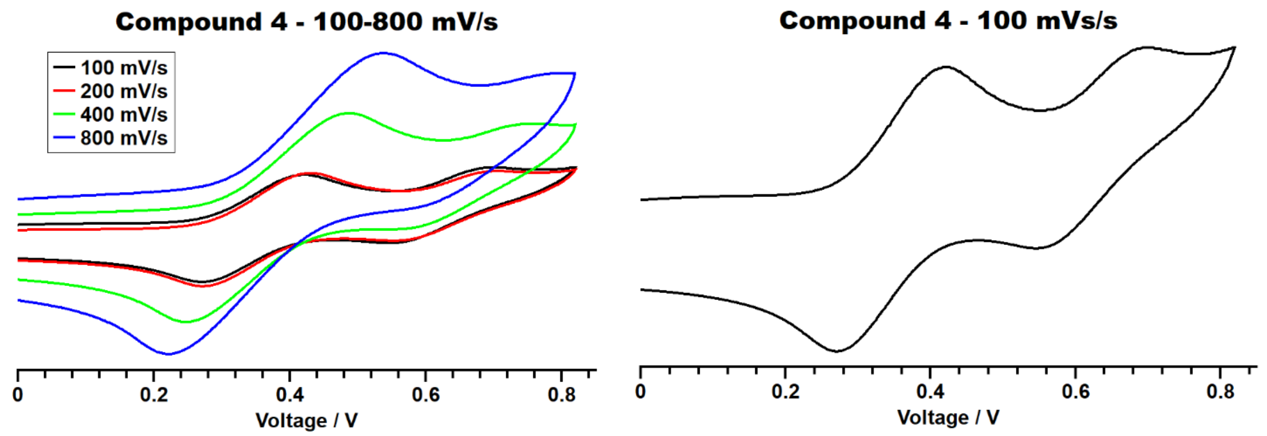


*Figure S 7 Cyclic voltammograms of compound 4 in dry DMSO/ 0.1 M $NBu_4PF_6$. Potentials are reported against ferrocene (Fc/Fc+ = 0.0 V).*

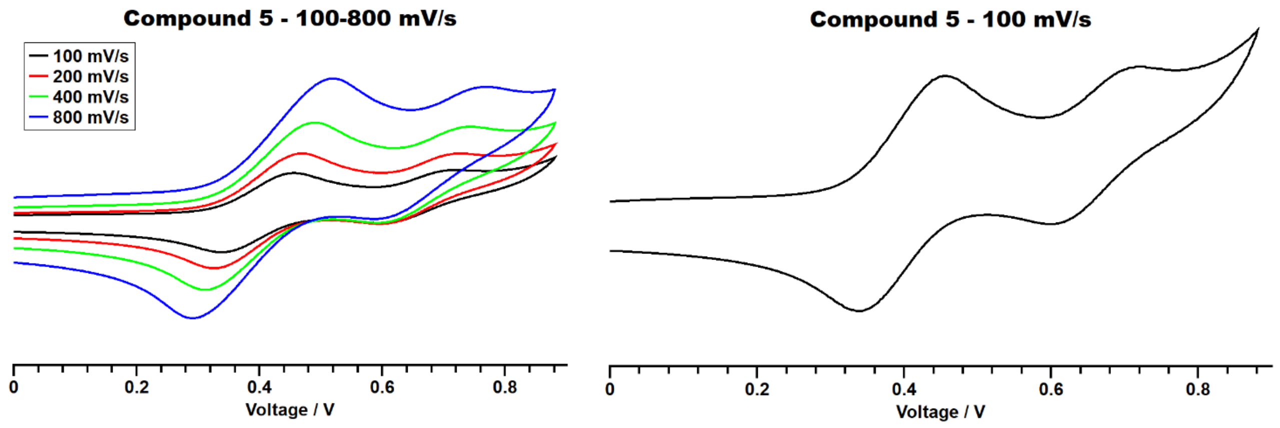


*Figure S 8 Cyclic voltammograms of compound **5** in dry DMSO/ 0.1 M $NBu_4PF_6$. Potentials are reported against ferrocene (Fc/Fc+ = 0.0 V).*

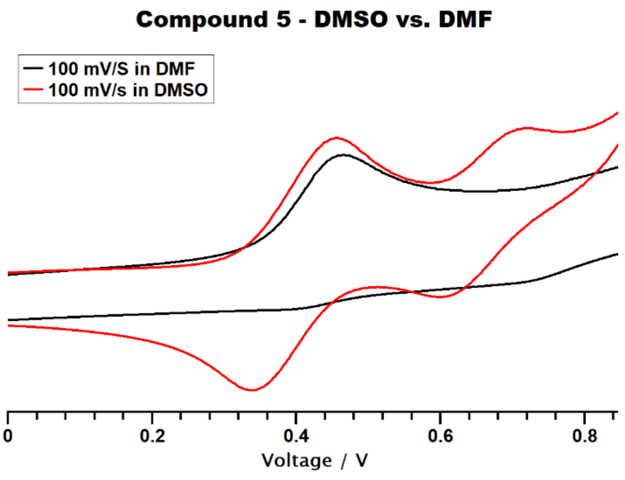


*Figure S 9. Cyclic voltammograms of compound **5** in either dry DMSO (red) or dry DMF (black)/ 0.1 M $NBu_4PF_6$. Potentials are reported against ferrocene (Fc/Fc+ = 0.0 V).*

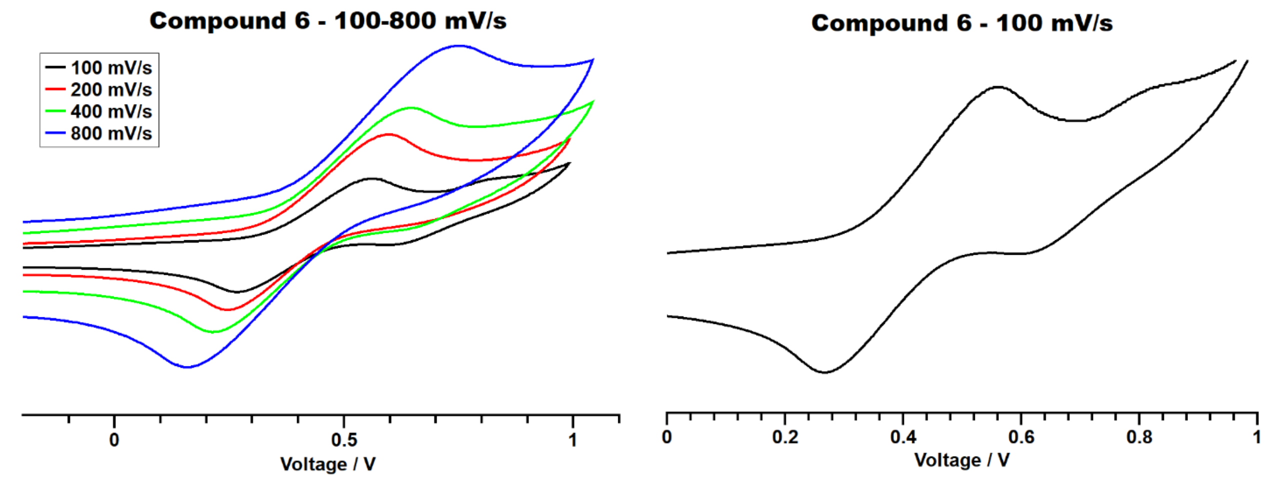


*Figure S 10. Cyclic voltammograms of compound 6 in dry DMSO/ 0.1* M *$NBu_4PF_6$. Potentials are reported against ferrocene (Fc/Fc+ = 0.0 V).*

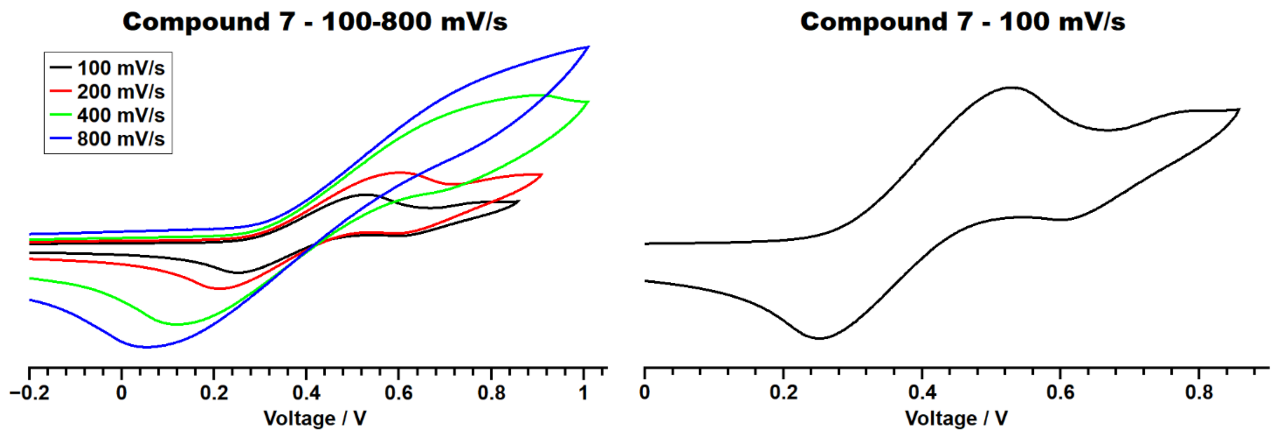


*Figure S 11. Cyclic voltammograms of compound 7 in dry DMSO/ 0.1* M *$NBu_4PF_6$. Potentials are reported against ferrocene (Fc/Fc+ = 0.0 V).*

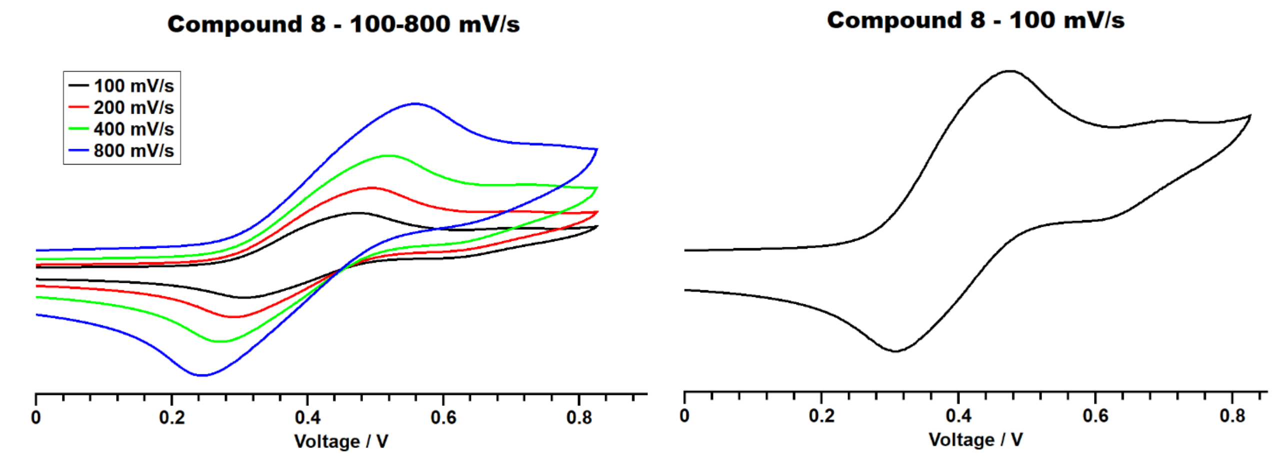


*Figure S 12. Cyclic voltammograms of compound 8 in dry DMSO/ 0.1* M *$NBu_4PF_6$. Potentials are reported against ferrocene (Fc/Fc+ = 0.0 V).*

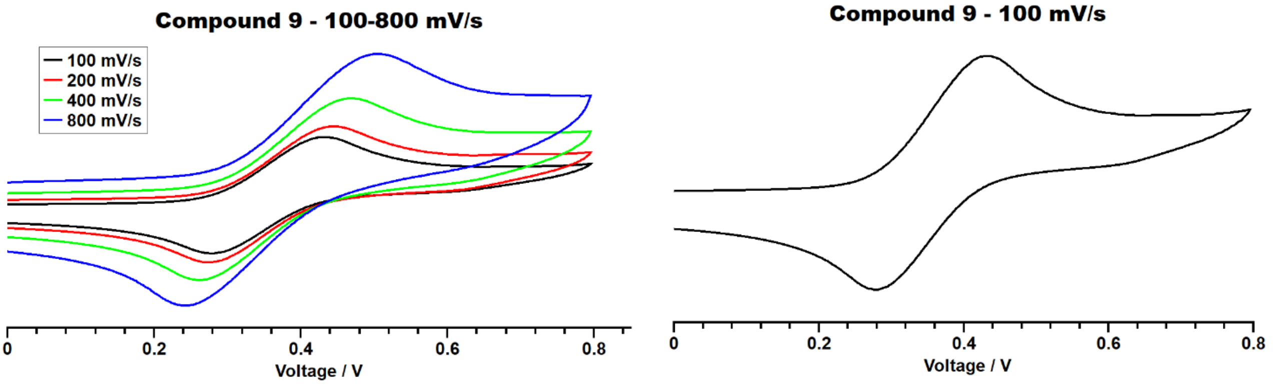


*Figure S 13. Cyclic voltammograms of compound 9 in dry DMSO/ 0.1 M $NBu_4PF_6$. Potentials are reported against ferrocene (Fc/Fc+ = 0.0 V).*

*Table S 1 Cyclic voltammetric data for compounds 1-9 in dry DMSO/ 0.1 M NBu4PF6; scan rate = 100 mV/s. Potentials are reported in V and $E_{1/2}$ (parenthesis) in mV. Referenced against ferrocene (Fc/Fc+ = 0.0 V).*

| **Compounds** | **$Epa_1$** | **$Epa_2$** | **$Epc_2$** | **$Epc_1$** | **$\Delta Ep_1$** ($E_{1/2}$) | **$\Delta Ep_2$** ($E_{1/2}$) |
|---|---|---|---|---|---|---|
| **$VO(acac)_2$** | 0.46 | 0.73 | 0.58 | 0.29 | 0.17 (85) | 0.15 (75) |
| **1** | 0.44 | 0.67 | 0.61 | 0.35 | 0.09 (45) | 0.06 (30) |
| **2** | 0.44 | 0.68 | 0.62 | 0.36 | 0.08 (40) | 0.06 (30) |
| **3** | 0.49 | 0.71 | 0.62 | 0.32 | 0.17 (85) | 0.09 (45) |
| **4** | 0.42 | 0.69 | 0.56 | 0.28 | 0.14 (70) | 0.13 (65) |
| **5** | 0.45 | 0.7 | 0.62 | 0.34 | 0.11 (55) | 0.08 (40) |
| **6** | 0.56 | 0.81 | 0.62 | 0.27 | 0.29 (145) | 0.19 (95) |
| **7** | 0.52 | 0.77 | 0.62 | 0.26 | 0.26 (130) | 0.15 (75) |
| **8** | 0.47 | 0.7 | 0.61 | 0.31 | 0.16 (80) | 0.09 (45) |
| **9** | 0.43 | ND | ND | 0.28 | 0.15 (75) | ND |

ND = not determined

# Cytotoxicity Studies

## $IC_{50}$ values

*Table S 2. Complete $IC_{50}$ values ($\mu M$) ± SD for cancer cell lines: lung (A549), colon (HCT116), cervical (HeLa), breast (MCF-7 and MDA-MB-231) and pancreatic (MIA PaCa-2), and normal cell line RPE-1, when exposed to compounds **1-9** for 48, 72 or 96 h ($IC_{50}$ values at 24 h were generally >100 $\mu M$). Errors represent the standard deviation from triplicate repeats.*

| | A549 | | | HCT-116 | | | HeLa | | | MCF-7 | | | MIA PaCa-2 | | | RPE-1 | |
|---|---|---|---|---|---|---|---|---|---|---|---|---|---|---|---|---|---|
| | **48h** | **72h** | **96h** | **48h** | **72h** | **96h** | **48h** | **72h** | **96h** | **48h** | **72h** | **96h** | **48h** | **72h** | **96h** | **72h** | **96h** |
| **1** | 26.0±5.2 | 21.2±0.1 | 22.5±0.3 | 44.4±3.2 | 43.5±2.1 | 40.8±2.3 | >100 | 82.8±2.7 | 72.0±1.8 | >100 | 82.2±7.1 | 66.8±0.1 | >100 | 58.2±3.5 | 38.8±1.1 | ND | |
| **2** | 6.9±2.1 | 7.9±0.9 | 7.5±1.2 | 39.0±4.6 | 31.3±4.3 | 32.4±2.4 | 88.8±0.7 | 91.5±1.8 | 75.7±1.7 | 91.9±0.5 | 37.3±1.3 | 32.1±2.1 | 82.0±1.8 | 15.1±0.1 | 20.2±0.1 | 41.2±2.1 | 38.6±4.1 |
| **3** | 7.2±0.5 | 6.9±0.9 | 7±1 | 51.9±1.8 | 50.4±1.3 | 43.7±0.3 | 56.3±5.5 | 90.8±4.1 | 77.6±4.2 | >100 | 48.6±2.8 | 37.2±1.3 | 87.0±1.5 | 22.6±0.2 | 22.1±0.8 | 37.6±1.1 | 39.4±0.2 |
| **4** | 13.5±1.7 | 10.7±0.1 | 11.1±0.9 | 48.9±3.0 | 49.8±2.8 | 45.9±1.5 | 74.4±3.7 | 86.1±8.2 | 72.7±2.1 | >100 | 70.0±8.8 | 45.4±1.1 | >100 | 28.8±6.8 | 30.5±7.8 | ND | |
| **5** | 6.5±0.6 | 7.5±0.1 | 8.0±0.4 | 40.7±5.5 | 42.1±0.3 | 40.2±1.5 | 96.4±3.5 | 89.1±2.6 | 75.3±1.5 | >100 | 63.2±0.4 | 45.9±0.8 | 95.6±2.9 | 44.41.5 | 27.0±3.2 | ND | |
| **6** | 5.7±0.6 | 6.8±0.9 | 7.6±0.4 | 50.8±3.5 | 41.6±2.7 | 36.7±1.7 | >100 | 84.6±2.2 | 83.3±0.5 | >100 | 61.0±3.7 | 42.0±0.3 | 96.3±0.3 | 43.4±0.2 | 22.2±0.7 | ND | |
| **7** | 6.1±0.2 | 8.7±0.6 | 8.2±0.3 | 48.0±7.9 | 42.3±2.3 | 39.9±0.2 | >100 | 83.6±0.8 | 74.4±3.2 | >100 | 67.4±0.9 | 44.1±0.5 | >100 | 44.9±1.0 | 24.0±0.5 | ND | |
| **8** | 8.6±0.4 | 7.6±0.2 | 8.7±0.2 | 45.9±3.5 | 47.1±0.2 | 40.5±3.0 | >100 | 47.9±3.1 | 40.1±0.6 | >100 | 81.5±0.3 | 55.4±0.9 | >100 | 49.5±0.7 | 24.3±0.1 | 38.1±0.4 | 36.6±0.9 |
| **9** | 7.5±2.8 | 11.0±0.6 | 10.1±0.7 | 59.0±0.8 | 39.2±0.3 | 19.9±0.2 | >100 | 46.0±0.6 | 36.3±0.4 | >100 | 23.9±4.6 | 6.7±3.3 | 76.4±1.4 | 16.7±0.2 | 18.1±2.5 | 33.3±2.0 | 38.1±1.7 |

## Microscope Images

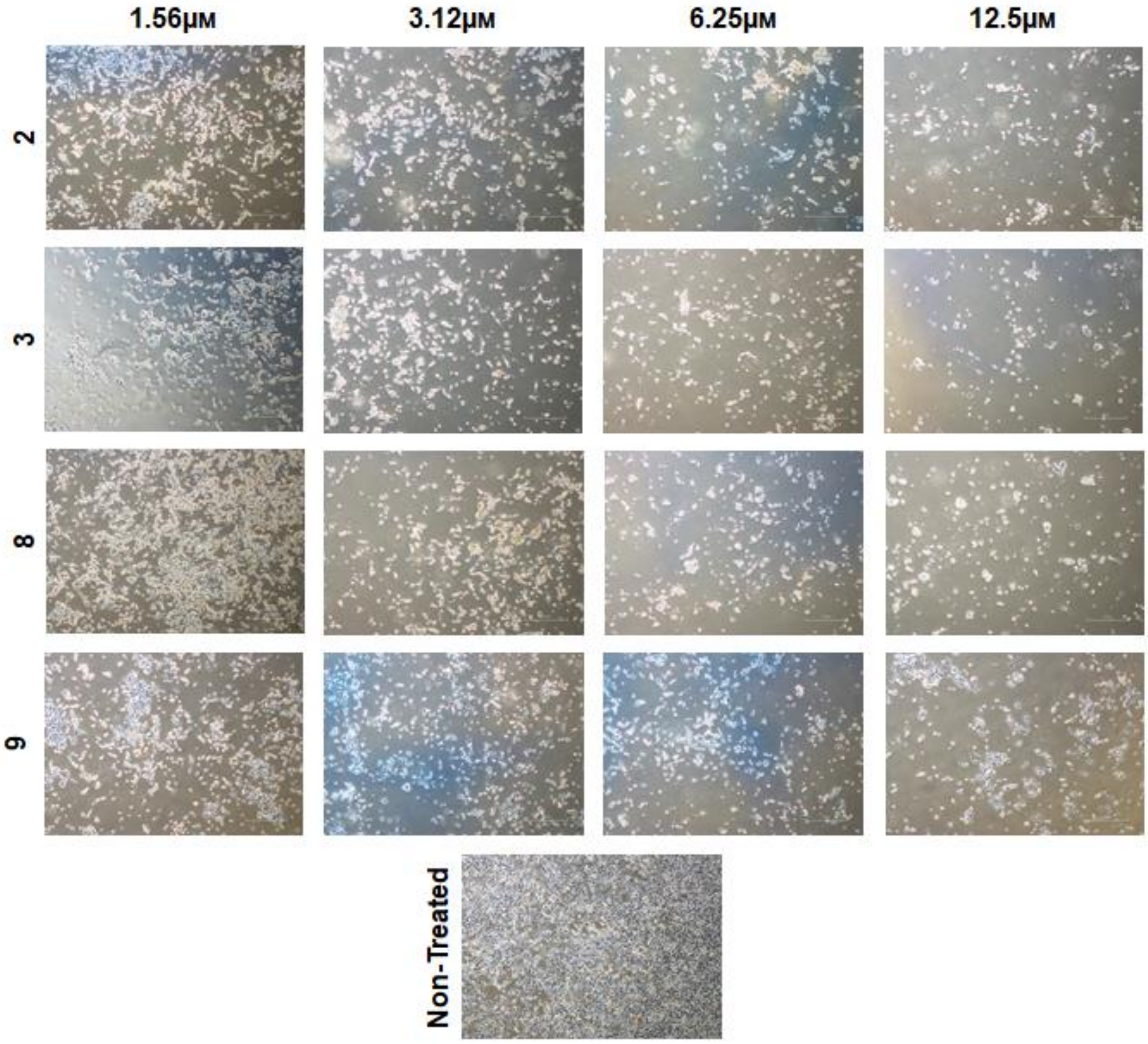


*Figure S 14. Microscope images of A549 cells after 72 h exposure to compounds **2**, **3**, **8** and **9**, at concentrations of 1.56, 3.12, 6.25 and 12.5 µM.*

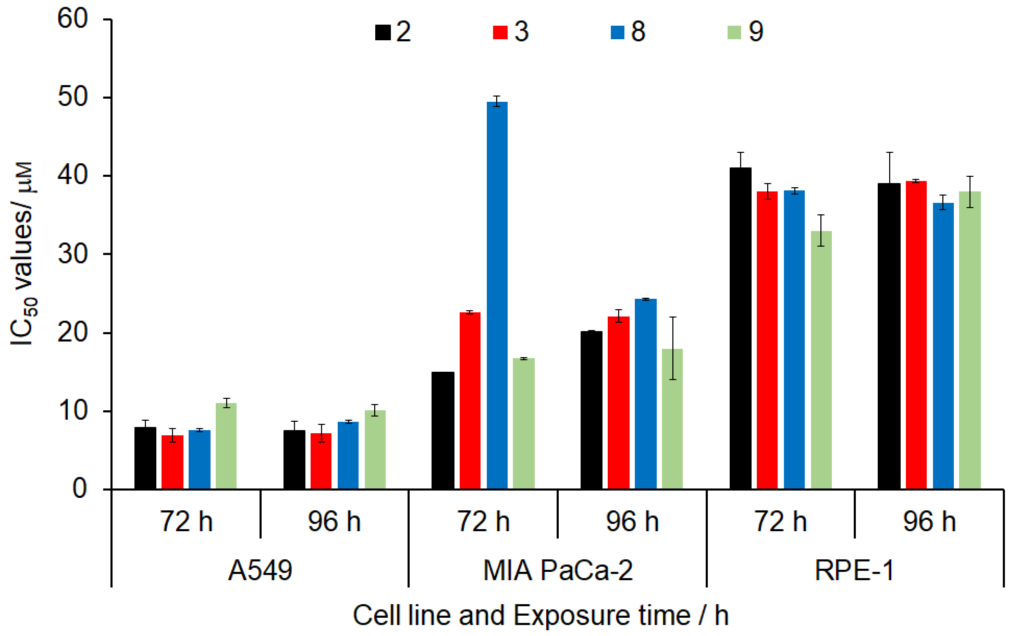


*Figure S 15. $IC_{50}$ values (µM) when cell lines A549, MIA PaCa-2 and RPE-1 are exposed to compounds **2**, **3**, **8** and **9** for 72 or 96 h.*

## Complex–DNA interaction by agarose gel mobility assay.

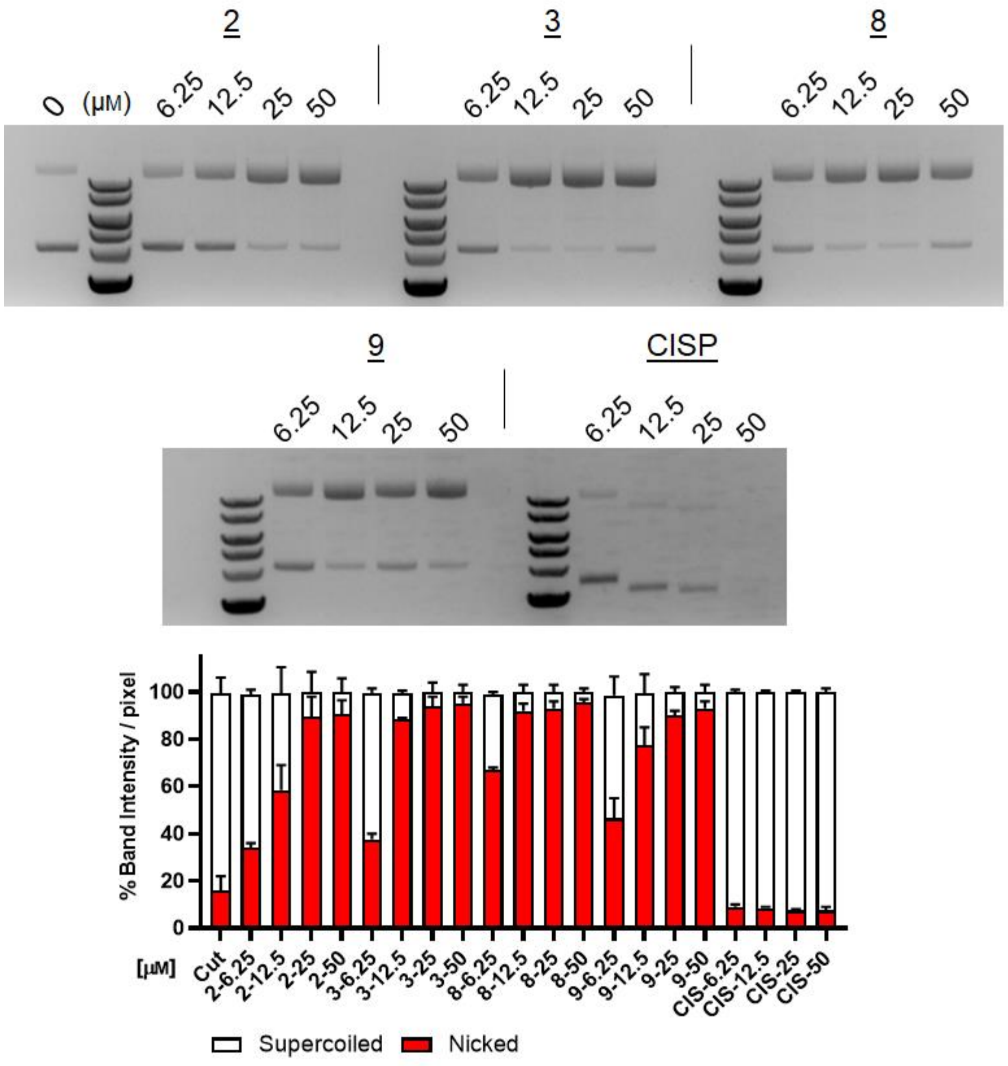


*Figure S 16. Gel electrophoresis In vitro analysis of compounds* ***2****,* ***3****,* ***8*** *and* ***9*** *and plasmid DNA. Plasmid DNA was incubated with different concentrations of compound (6.25 to 50 μM) for 24 h at room temperature. The intensity of the DNA bands in each lane was quantified and represented as mean ± SD (n = 2) for nicked circular and super coiled.*

# FRET and FID Biophysical Measurements

## Raw Data for Compounds 1-9 at 1 μM (5 eq.)

### Compound 1

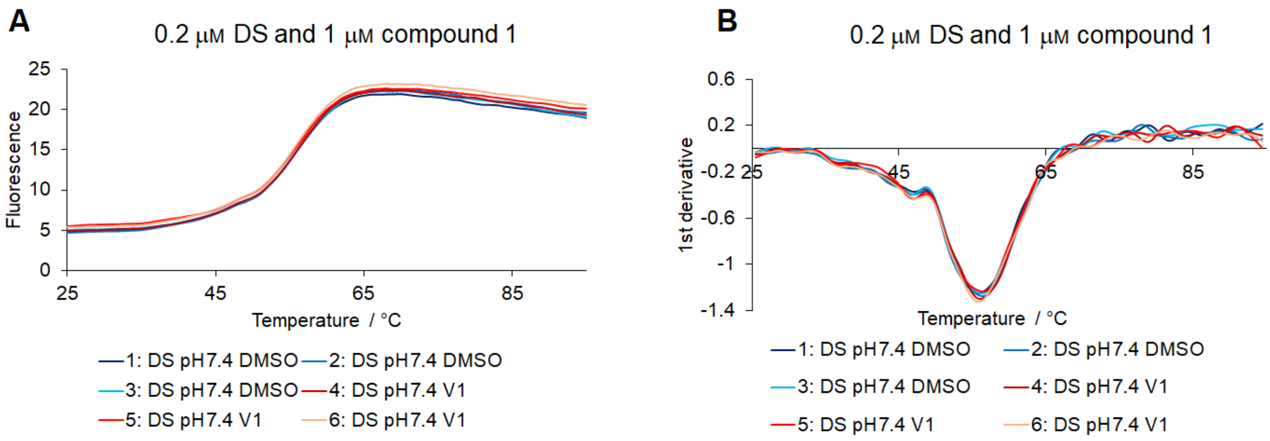


*Figure S 17. Fluorescence melting graphs (A) and 1st derivatives (B) when screened with 1 μM compound **1** and 0.2 μM DS DNA is heated between 25-90 °C (10 mM NaCaco, 100 mM KCl and pH 7.4, blue = DMSO repeats, red/orange = compound **1** repeats)*

### Compound 2

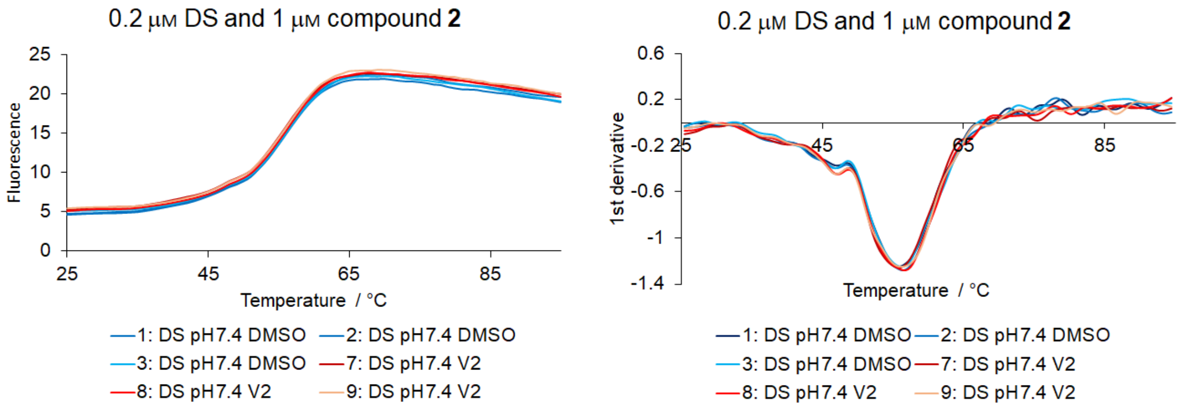


*Figure S 18. Fluorescence melting graphs (A) and 1st derivatives (B) when screened with 1 μM compound **2** and 0.2 μM DS DNA is heated between 25-90 °C (10 mM NaCaco, 100 mM KCl and pH 7.4, blue = DMSO repeats, red/orange = compound **2** repeats)*

## Compound 3

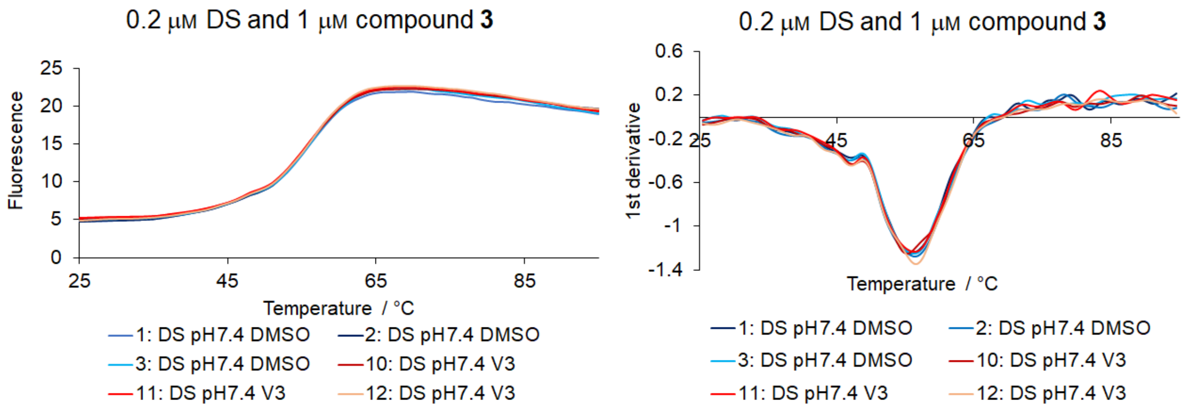


*Figure S 19. Fluorescence melting graphs (A) and 1st derivatives (B) when screened with 1 μM compound **3** and 0.2 μM DS DNA is heated between 25-90 °C (10 mM NaCaco, 100 mM KCl and pH 7.4, blue = DMSO repeats, red/orange = compound **3** repeats)*

## Compound 4

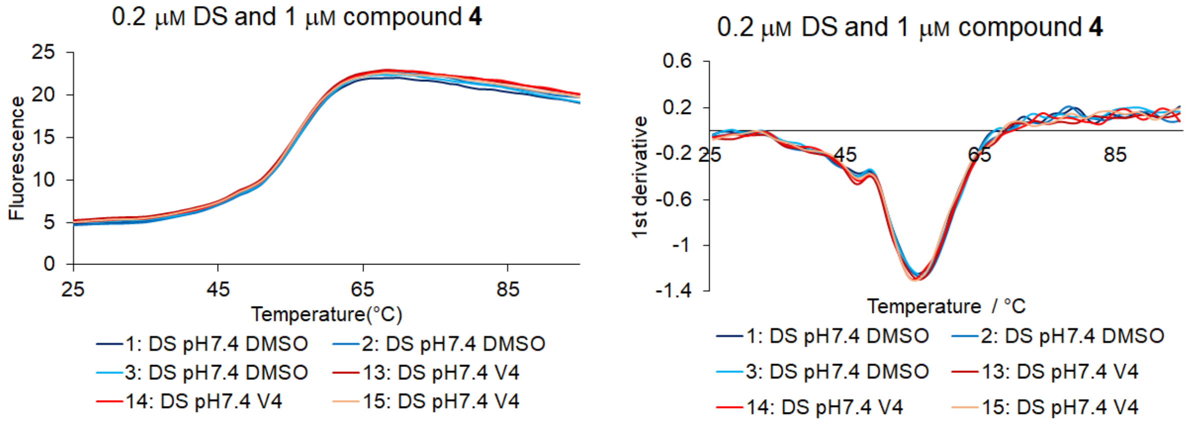


*Figure S 20. Fluorescence melting graphs (A) and 1st derivatives (B) when screened with 1 μM compound **4** and 0.2 μM DS DNA is heated between 25-90 °C (10 mM NaCaco, 100 mM KCl and pH 7.4, blue = DMSO repeats, red/orange = compound **4** repeats)*

## Compound 5

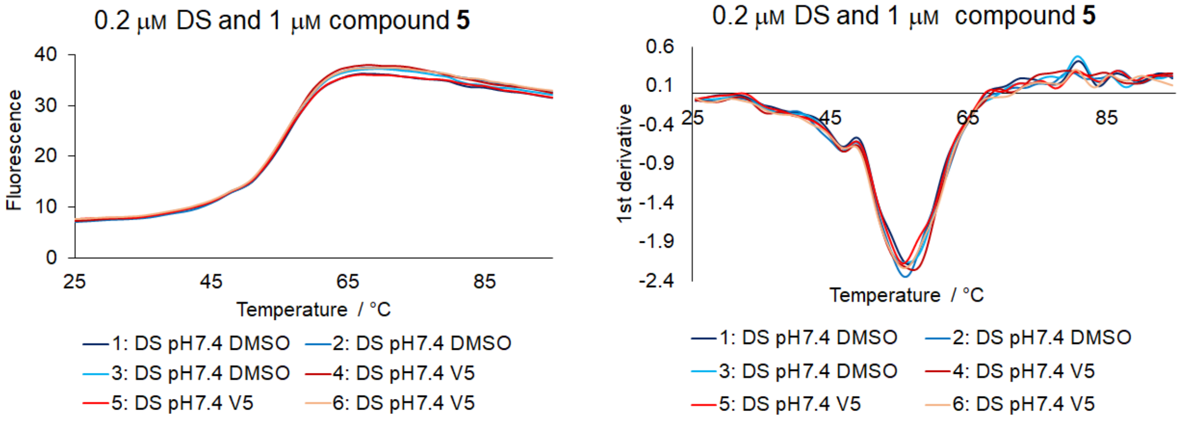


*Figure S 21 Fluorescence melting graphs (A) and 1st derivatives (B) when screened with 1 μM compound **5** and 0.2 μM DS DNA is heated between 25-90 °C (10 mM NaCaco, 100 mM KCl and pH 7.4, blue = DMSO repeats, red/orange = compound 5 repeats)*

## Compound 6

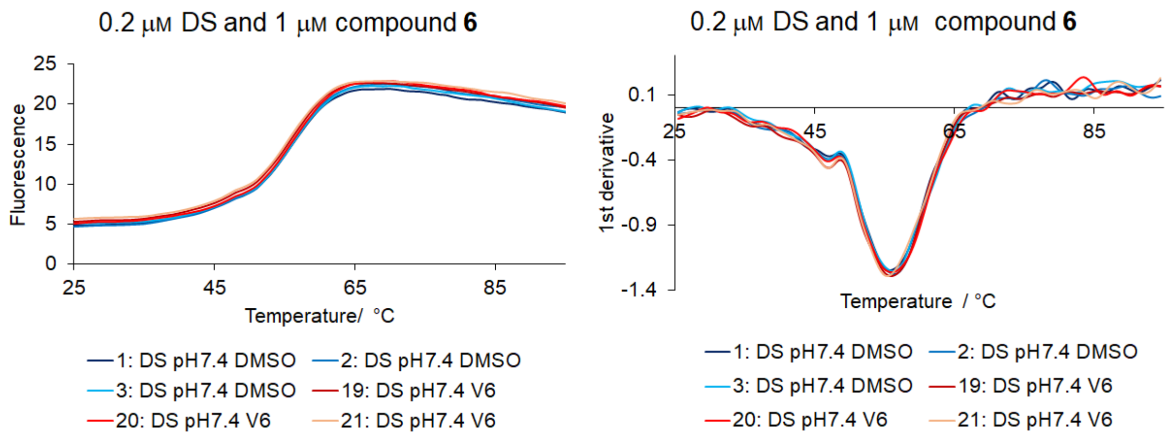


*Figure S 22 Fluorescence melting graphs (A) and 1st derivatives (B) when screened with 1 μM compound **6** and 0.2 μM DS DNA is heated between 25-90 °C (10 mM NaCaco, 100 mM KCl and pH 7.4, blue = DMSO repeats, red/orange = compound **6** repeats)*

## Compound 7

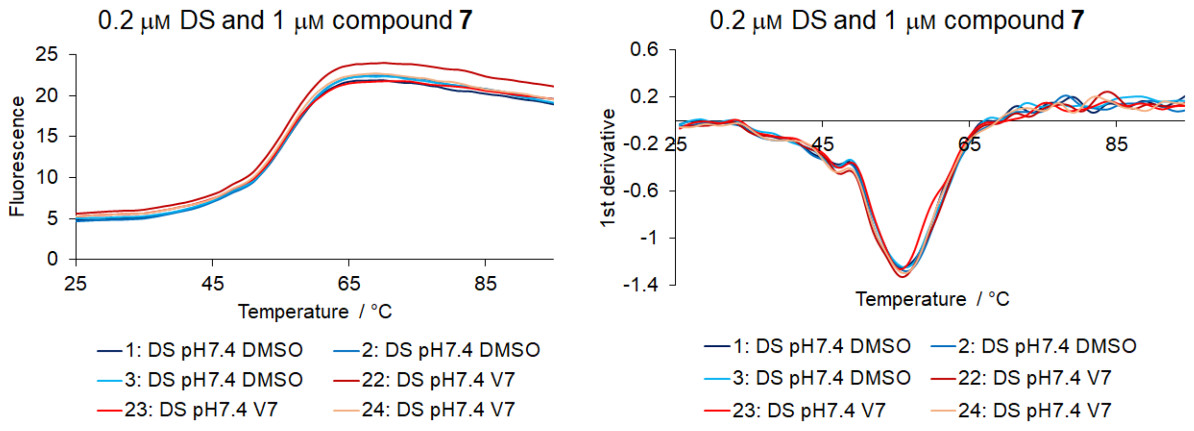


*Figure S 23 Fluorescence melting graphs (A) and 1st derivatives (B) when screened with 1 μM compound **7** and 0.2 μM DS DNA is heated between 25-90 °C (10 mM NaCaco, 100 mM KCl and pH 7.4, blue = DMSO repeats, red/orange = compound **7** repeats)*

## Compound 8

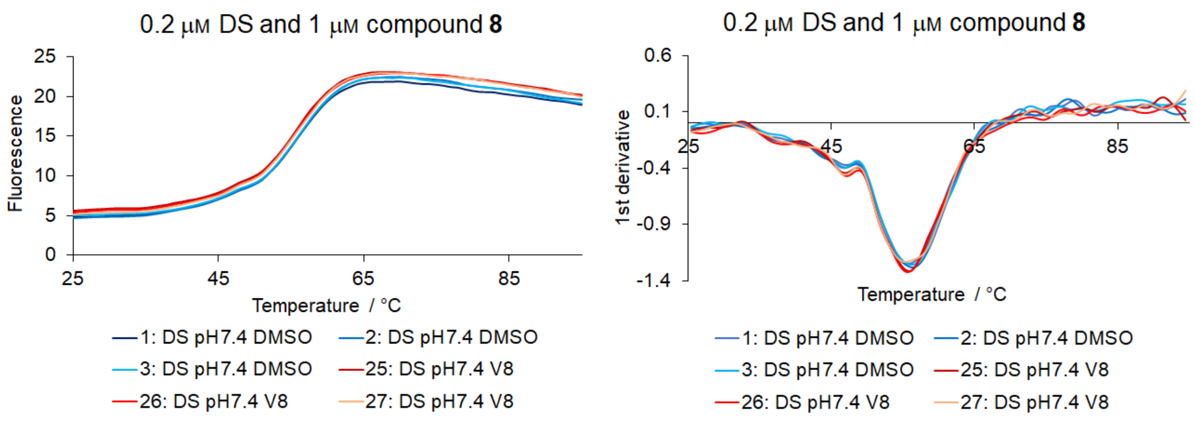


*Figure S 24 Fluorescence melting graphs (A) and 1st derivatives (B) when screened with 1 μM compound **8** and 0.2 μM DS DNA is heated between 25-90 °C (10 mM NaCaco, 100 mM KCl and pH 7.4, blue = DMSO repeats, red/orange = compound 8 repeats)*

Compound 9

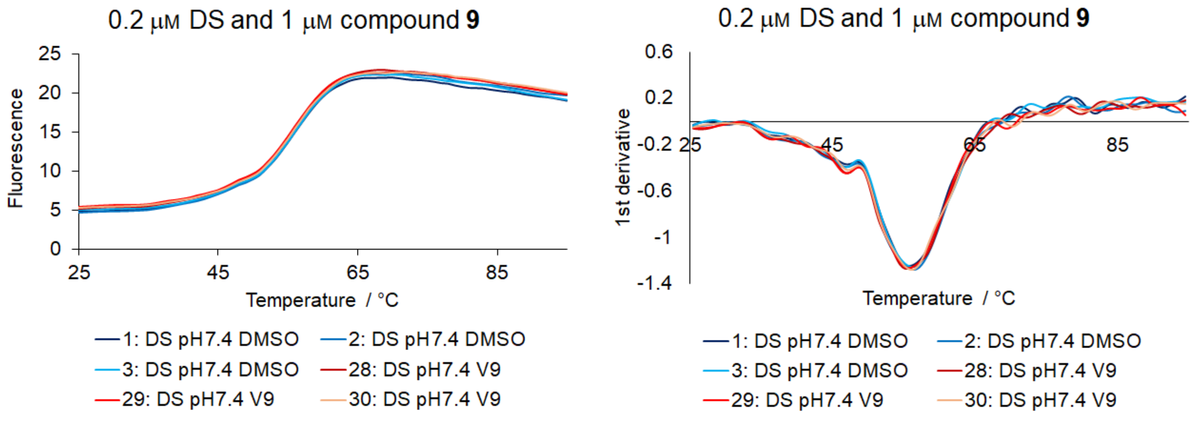


*Figure S 25 Fluorescence melting graphs (A) and 1st derivatives (B) when screened with 1 μM compound **9** and 0.2 μM DS DNA is heated between 25-90 °C (10 mM NaCaco, 100 mM KCl and pH 7.4, blue = DMSO repeats, red/orange = compound 9 repeats)*

## Results with 25 μM of test compound

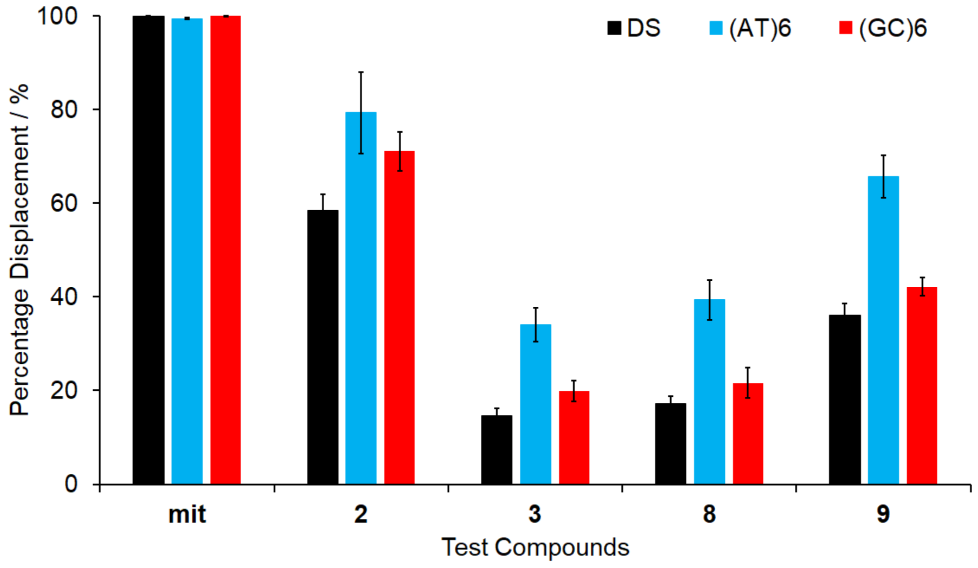


*Figure S 26 Percentage displacements (%) for 25 μM (25 eq) of mitoxantrone (mit, positive control) and compounds **2**, **3**, **8** and **9**; black = DS, blue = $(AT)_6$ and red = $(GC)_6$ (all at 1 μM).*

*Table S 3 FRET melting and FID data with DS, $(AT)_6$, $(GC)_6$ and 25 µM of compound. FRET melting $\Delta T_m$ with DS DNA (0.2 µM) and compounds **2**, **3**, **8**, **9** (25 µM, 125 eq.). FID % TO displacement of 25 µM (25 eq) complex with 1 µM of a DNA. Buffer: 100 mM potassium chloride and 10 mM sodium cacodylate at pH 7.4*

| **Test compounds** | **FRET Melting $\Delta T_m$ (°C)** | **TO Displacement, $D_{TO}$ (%)** | | |
|---|---|---|---|---|
| | | **DS** | **$(AT)_6$** | **$(GC)_6$** |
| **mitoxantrone** | -- | 100±1 | 99±2 | 100±1 |
| **2** | 0.3±0.2 | 59±3 | 79±9 | 71±4 |
| **3** | 0.7±1.0 | 15±2 | 34±4 | 20±2 |
| **8** | 0.4±0.3 | 17±2 | 39±4 | 22±3 |
| **9** | 0.0±0.2 | 36±3 | 66±5 | 42±2 |

## Raw Data for Compounds 2, 3, 8 and 9 at 25 µM (125 eq.)

### Compound 2

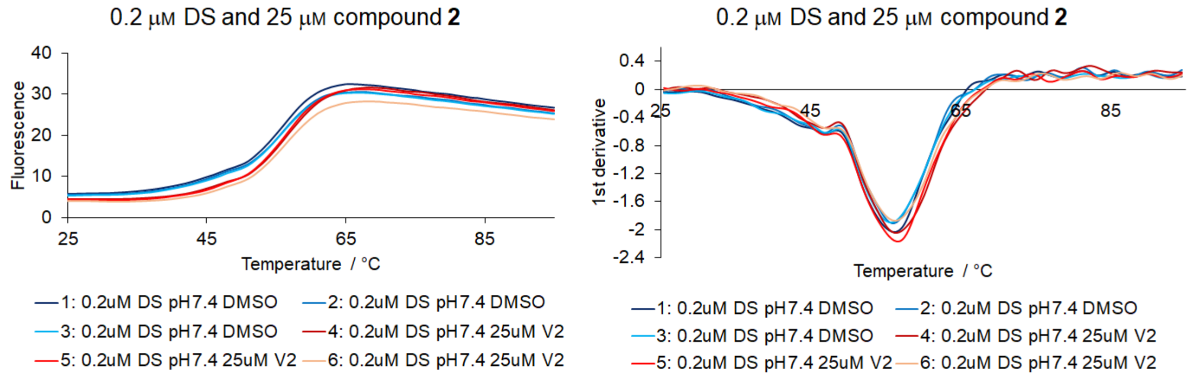


*Figure S 27 Fluorescence melting graphs (A) and 1st derivatives (B) when screened with 25 µM compound **2** and 0.2 µM DS DNA is heated between 25-90 °C (10 mM NaCaco, 100 mM KCl and pH 7.4, blue = DMSO repeats, red/orange = compound **9** repeats)*

### Compound 3

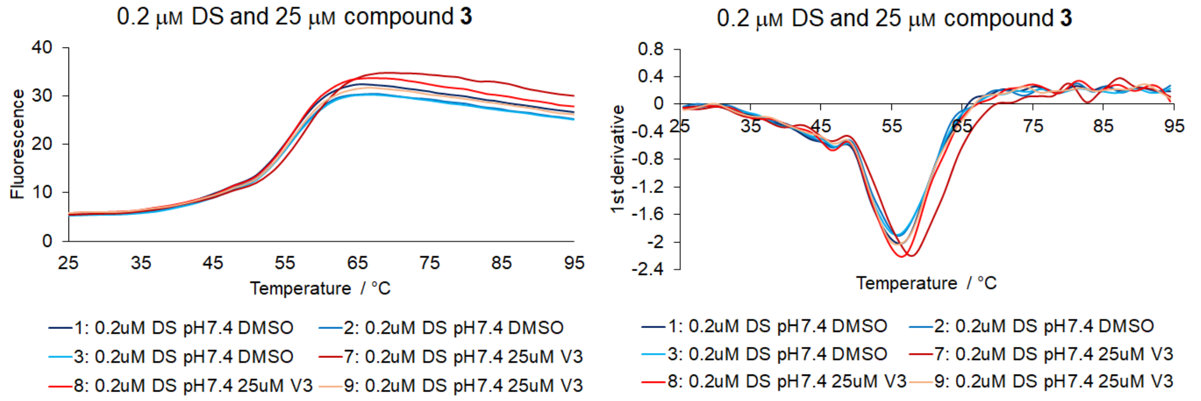


*Figure S 28 Fluorescence melting graphs (A) and 1st derivatives (B) when with screened 25 µM compound **3** and 0.2 µM DS DNA is heated between 25-90 °C (10 mM NaCaco, 100 mM KCl and pH 7.4, blue = DMSO repeats, red/orange = compound **9** repeats)*

## Compound 8

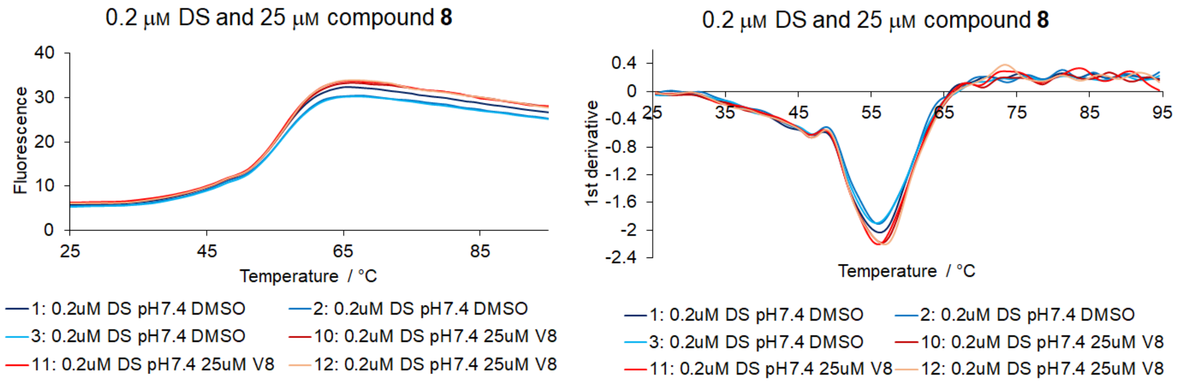


*Figure S 29 Fluorescence melting graphs (A) and 1st derivatives (B) when with screened 25 µM compound **8** and 0.2 µM DS DNA is heated between 25-90 °C (10 mM NaCaco, 100 mM KCl and pH 7.4, blue = DMSO repeats, red/orange = compound **9** repeats)*

## Compound 9

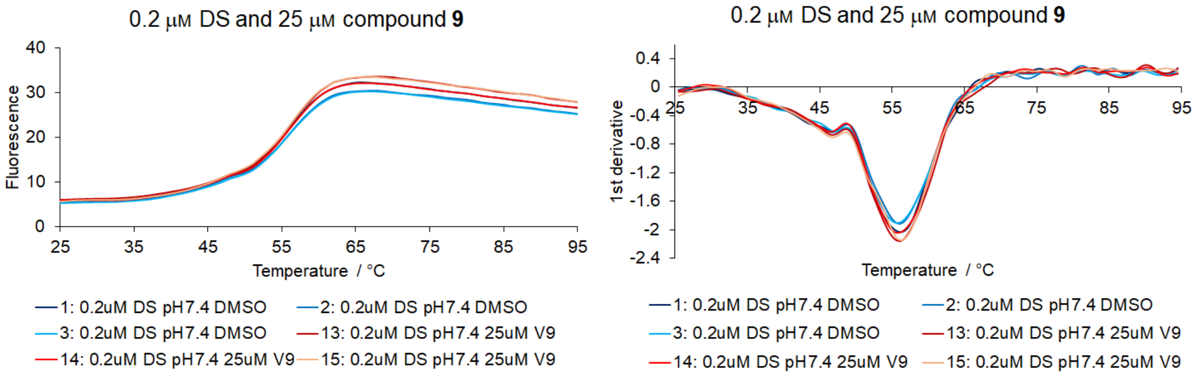


*Figure S 30 Fluorescence melting graphs (A) and 1st derivatives (B) when with screened 25 µM compound **3** and 0.2 µM DS DNA is heated between 25-90 °C (10 mM NaCaco, 100 mM KCl and pH 7.4, blue = DMSO repeats, red/orange = compound **9** repeats)*

## Apoptosis and Caspase-3/7

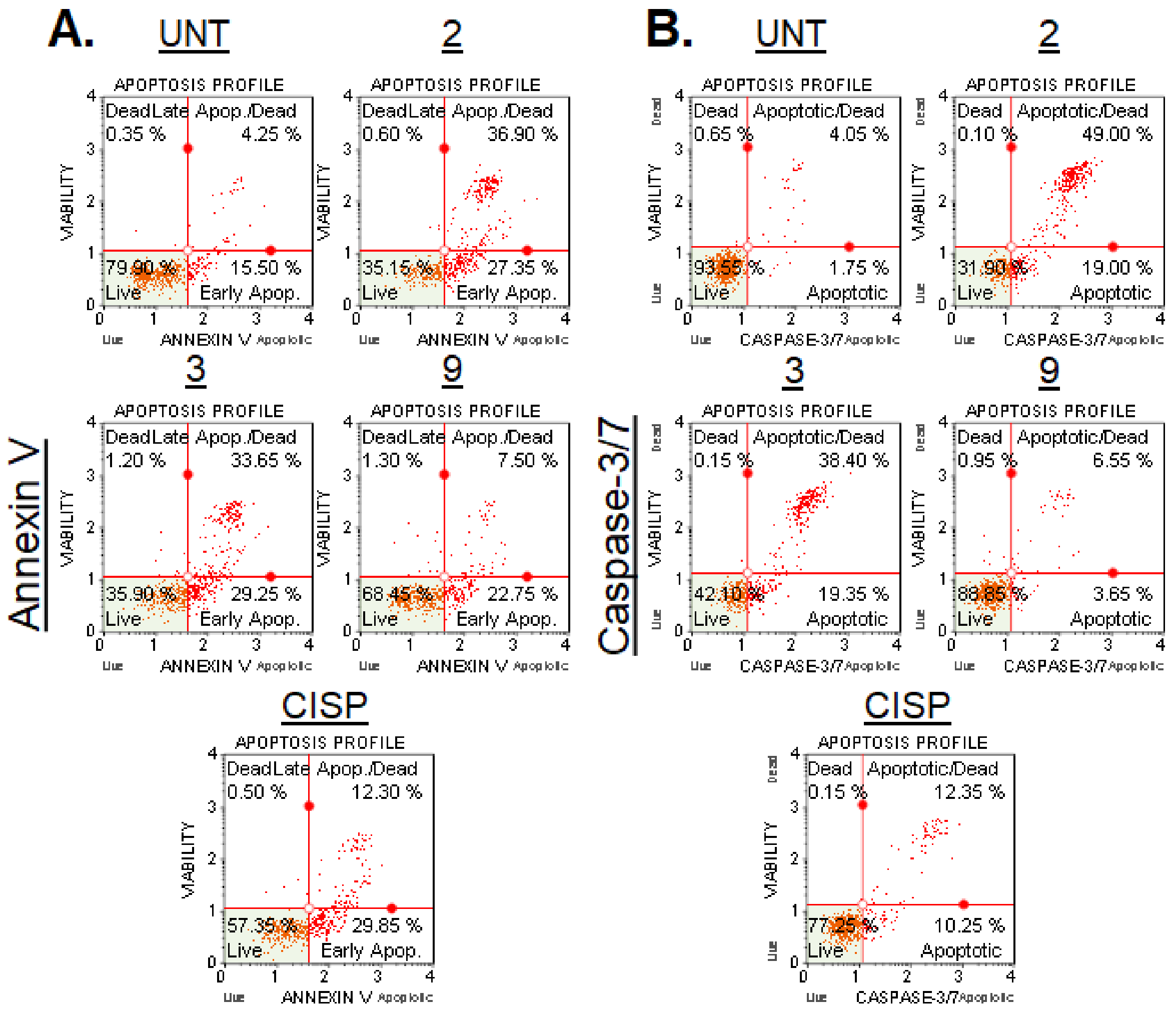


*Figure S 31 A. Flow cytometric analysis using Annexin-V staining with 7-AAD incorporation; B. Flow cytometric analysis of the activated Caspase-3/7 and simultaneous detection of dead cells by 7-AAD staining. In both cases, A549 cells were exposed to compounds **2**, **3**, **8** and **9** (25 μM) for 48 h. Percentages of cells in quadrant are given as non-apoptotic live (lower left), non-apoptotic dead (upper left), early apoptotic (lower right), and late-apoptotic (upper right).*
